# Peptide‐Bound Aflibercept Eye Drops for Treatment of Neovascular Age‐Related Macular Degeneration in Nonhuman Primates

**DOI:** 10.1002/advs.202410744

**Published:** 2025-01-30

**Authors:** Xingyan Fan, Kuan Jiang, Yongqian Zhao, Benjamin TK Lee, Feiyang Geng, Marten E Brelen, Weiyue Lu, Gang Wei

**Affiliations:** ^1^ Department of Pharmaceutics School of Pharmacy Fudan University & Key Laboratory of Smart Drug Delivery (Fudan University) Ministry of Education Shanghai 201203 China; ^2^ Eye Institute and Department of Ophthalmology Eye and ENT Hospital Fudan University Shanghai 200031 China; ^3^ Alephoson Biopharmaceuticals Limited Hong Kong SAR 999077 China; ^4^ Department of Ophthalmology and Visual Sciences The Chinese University of Hong Kong Hong Kong SAR 999077 China; ^5^ Quzhou Fudan Institute Quzhou 324003 China; ^6^ Shanghai Engineering Research Center of ImmunoTherapeutics Shanghai 201203 China

**Keywords:** aflibercept, age‐related macular degeneration, cell‐penetrating peptide, eye drops, hydrophobic interaction

## Abstract

The advent of biomacromolecules antagonizing vascular endothelial growth factor (VEGF) has revolutionized the treatment of neovascular age‐related macular degeneration (nAMD). However, frequent intravitreal injections of these biomacromolecules impose an enormous burden on patients and create a massive workload for healthcare providers. This causes patients to abandon therapy, ultimately leading to progressive and irreversible vision loss. In order to address this unmet clinical need, a noninvasive treatment for nAMD is developed. An optimized cell‐penetrating peptide derivative, ^bxy^Penetratin (bxyWP), is used to non‐covalently complex with the anti‐VEGF protein aflibercept (AFL) via reversible hydrophobic interaction. The interaction is crucial for AFL delivery, neither impairing the affinity of AFL to pathological VEGF, nor being interfered by endogenous proteins in tear fluids. AFL/bxyWP eye drops exhibit prolonged retention on the eye and excellent absorption into the posterior ocular segment following topical administration, with significant drug distribution to the retina and choroid. In a laser‐induced choroidal neovascularization model on cynomolgus monkeys, AFL/bxyWP eye drops efficiently reduce lesion size and leakage comparable to conventional intravitreal injection of AFL. These results suggest that AFL/bxyWP eye drops are feasible self‐administered treatment for neovascular retinal diseases and potentially become a substitute for intravitreal injections.

## Graphical abstract

This article demonstrates that the peptide ^bxy^Penetratin (bxyWP) non‐covalently binds to the anti‐vascular endothelial growth factor (VEGF) protein aflibercept via hydrophobic interactions, enabling noninvasive delivery of aflibercept to the choroid and retina through conjunctival‐scleral pathway and effective inhibition of choroidal neovascularization in cynomolgus monkeys.

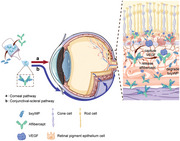

## 1 Introduction

Age‐related macular degeneration (AMD) is a disease characterized by drusen deposits, retinal pigment epithelial changes and, in the advanced stages, geographic atrophy or neovascular maculopathy.^[^ ^1^, ^2^ ^]^ AMD affects ≈196 million people globally,^[^ ^3^ ^]^ and it is estimated that by 2040, this number will increase to 288 million.^[^ ^4^ ^]^ Neovascular AMD (nAMD) is characterized by the growth of abnormal choroidal vessels into the subretinal space. These newly formed vessels tend to leak fluid or bleed, resulting in retinal edema or submacular haemorrhage. Patients experience metamorphopsia and blurring of vision and, if left untreated, permanent sight loss. Untreated nAMD results in severe visual impairment with an average loss of 4 lines of visual acuity within 2 years of disease onset.^[^ ^5^ ^]^

The development of choroidal neovascularization (CNV) in advanced nAMD is predominantly driven by pathological vascular endothelial growth factor (VEGF). VEGF is a potent mitogen for endothelial cells that increases vascular permeability and promotes angiogenesis.^[^ ^6^ ^]^ Targeting VEGF by intravitreally administered anti‐VEGF therapies, such as bevacizumab, ranibizumab, aflibercept (AFL), brolucizumab, or faricimab, has already been shown in large multicenter randomized controlled clinical trials to be a safe and efficacious treatment of nAMD.^[^ ^7^, ^8^, ^9^ ^]^ With prompt treatment, anti‐VEGF therapy stabilizes the vision in 90% of cases and improves vision by 2 lines of visual acuity in one‐third of patients. Since the widespread use of anti‐VEGF to treat patients with nAMD, the number of blind registrations due to AMD has dramatically fallen.^[^ ^10^ ^]^ However, despite the advances in anti‐VEGF treatments, nAMD remains one of the most common causes of blindness in the world.^[^ ^11^ ^]^

There are several theories on why people fail treatment with intravitreal anti‐VEGF treatment. One suggestion is that patients stop treatment early due to the burden of multiple intravitreal injections. Patients with nAMD require regular injections to stabilize their disease, on average, 6 injections in the first year and 4 or 5 injections in the second year.^[^ ^12^ ^]^ This considerable treatment burden to both the patient and the healthcare profession is one of the main reasons why treatments are stopped early, resulting in suboptimal outcomes. This also explains why real‐world outcomes of nAMD treatment are usually worse than the results from clinical trials.^[^ ^13^ ^]^ Therefore, a more convenient and less invasive treatment strategy is imperatively needed to improve these treatment outcomes.

Numerous physical barriers of the eye prevent drugs from reaching the retina by topical application, including the tear film, cornea, conjunctiva, and sclera. Distribution to the posterior segment via systemic administration is also impeded by the blood‐ocular barrier. Various penetration enhancers have been developed to promote drug absorption to the posterior segment after topical application, with cell‐penetrating peptides (CPPs) being one of the most frequently reported in the literature.^[^ ^14^ ^]^ CPPs are short‐chain peptides (< 30 amino acid residues) that can be easily internalized across cell membranes. More importantly, they can transport a variety of covalently or non‐covalently bonded drug molecules into living cells with negligible cytotoxicity.^[^ ^15^ ^]^ The CPP candidates that showed the potential to improve absorption in the posterior segment of the eye include transactivator of transcription (TAT), polyarginine (for example, R_8_), and penetratin (PE).^[^ ^16^ ^]^ However, the underlying mechanism by which CPP promotes drug delivery from the ocular surface to the retina has not yet been elucidated.

In recent years, CPPs have been widely explored in various biomedical applications to deliver macromolecules by forming intermolecular interactions, such as hydrophobic and electrostatic interactions and hydrogen bonding.^[^ ^17^, ^18^, ^19^ ^]^ For instance, RTP004, a highly positively charged peptide with a protein transduction domain, could form a strong electrostatic bond with botulinum toxin type A. This non‐covalent interaction enhanced binding of the neurotoxin to neuronal surfaces, increased the likelihood of neurotoxin internalization, and eventually improved both efficacy outcomes and duration of effect.^[^ ^20^ ^]^ Based on this principle, a product for intramuscular injection (DAXXIFY) successfully gained approval from US Food and Drug Administration (FDA) in 2022 for the temporary improvement in the appearance of moderate to severe glabellar lines in adult patients. For the treatment of diabetes, Diedrichsen et al. revealed the importance of intermolecular interaction on the improvement of insulin absorption using penetratin and its analogs.^[^ ^21^, ^22^ ^]^ Transmucosal delivery of proteins using CPPs has shown encouraging progress,^[^ ^23^ ^]^ but still lacks outstanding performance in the treatment of neovascular eye diseases. Intensive studies on the non‐covalent interactions can provide insightful information from the theoretical perspective to reveal the ocular delivery mechanism of anti‐VEGF proteins mediated by CPPs and promote the development of non‐invasive therapeutic regimen for nAMD.

We have previously used wild‐type penetratin as a template, preserving its basic amino acid sequences, to generate a series of derived peptides through hydrophobic amino acid mutations,^[^ ^24^ ^]^ which provide a favorable condition to understand the non‐covalent delivery mechanism of protein therapeutics. Here we propose and demonstrate that among these penetratin derivatives (Table S1, Supporting Information), ^bxy^Penetratin (tryptophan mutation of amino acid residues designated as b, x, and y in wild‐type penetratin, bxyWP) exhibits the strongest affinity for VEGF trap protein aflibercept (molecular weight 115 kD), and the greatest ocular penetration capability. We prove that although both bxyWP and aflibercept possess positive charges in physiological conditions, they could still bind via hydrophobic interaction to form the non‐covalent complex, which was stable in tear fluids without interference from endogenous proteins. With the assistance of bxyWP, the ocular retention of aflibercept was prolonged, and the absorption in the retina and choroid was significantly improved through a non‐corneal route. Consequently, administering AFL/bxyWP eye drops for 2 weeks demonstrated a therapeutic effect comparable to a single intravitreal injection of aflibercept in a nonhuman primate nAMD model.

## 2 Results

### 2.1 Cellular Uptake of Aflibercept Positively Correlates with the Binding Affinity Between Aflibercept and CPPs

**Figure 1:**
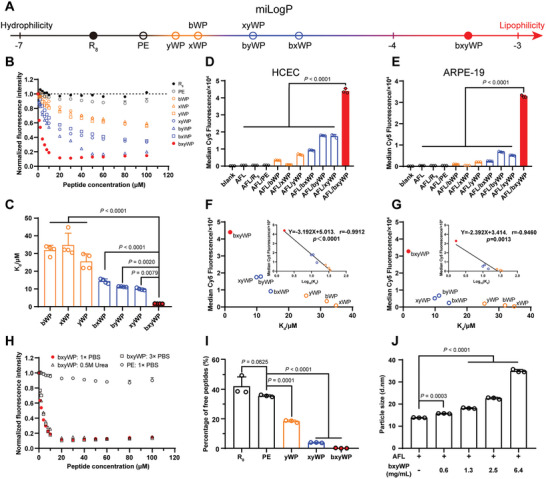
Interactions between aflibercept and CPPs. A) The oil‐water partition coefficient (miLogP) of CPPs was calculated using Molinspiration indicating their hydrophobicity. B,C) The dissociation constants (K_d_) for AFL/CPP complexes were determined using an equilibrium fluorescence quenching assay at 25 °C in 1 × PBS (*n* = 4). D,E) The quantitative cellular uptake of aflibercept promoted by different peptides was evaluated in human corneal epithelial cells (HCEC) and human retinal pigment epithelial cells (ARPE‐19). The AFL/CPP complexes (each containing 1 µm Cy5‐labelled aflibercept) were incubated with the cells for 2 h at 37 °C (*n* = 3). F,G) Correlation analysis was performed using linear regression between the K_d_ of the different penetratin‐derived peptides and aflibercept, and their ability to mediate aflibercept cellular uptake in HCEC and ARPE‐19 cells. H) The K_d_ for AFL/bxyWP complex was determined using equilibrium fluorescence quenching assay at 25 °C in 1× PBS with or without 0.5 m urea, or in 3× PBS (*n* = 4). I) The amount of unbound peptide in AFL/CPP complexes at the same molar concentration was determined using ultrafiltration (*n* = 3). J) The particle size of AFL/bxyWP complexes formed by 30 mg mL^−1^ aflibercept and different concentrations of bxyWP were measured (*n* = 3). Data are presented as means ± SD. Statistical analysis was performed using one‐way ANOVA with multiple comparisons corrected by Dunnett's test in (C‐E), (I), and (J).

A series of peptide derivatives with varying hydrophobicity were derived via amino acid mutation from wild‐type penetratin (**Figure** **1**A), and their binding pattern with aflibercept was studied. According to Figure 1B,C, the binding affinity between the penetratin derivatives and aflibercept was positively correlated with the degree of peptide hydrophobicity. The highest affinity was observed for the most hydrophobic bxyWP, with a dissociation constant (K_d_) of 1.66 ± 0.04 µm, significantly lower than the other counterparts. Besides, bxyWP showed the strongest ability to promote the cellular uptake of Cyanine 5 (Cy5)‐labelled aflibercept in both human corneal epithelial cells (HCEC) and human retinal pigment epithelial cells (ARPE‐19) (Figure 1D,E). In HCEC, the median fluorescence intensity of AFL/bxyWP complex treated cells was 108‐fold and 94‐fold higher than that of free aflibercept or AFL/PE treated cells, respectively. In ARPE‐19 cells, the corresponding increases were 101‐fold and 96‐fold. There was a strong linear correlation between cellular uptake and Log_10_(K_d_) (Figure 1F,G), as the stronger the interaction between the peptide and aflibercept (with lower K_d_), the more effective it was for the peptide to facilitate the cellular uptake of aflibercept.

Compared to wild‐type penetratin, the binding affinity of bxyWP with aflibercept sharply increased due to its enhanced hydrophobicity. Notably, the binding affinity between bxyWP and aflibercept was only slightly disrupted by 0.5 m urea and high salt concentration (Figure 1H; Figure S1, Supporting Information), revealing hydrogen binding or electrostatic interaction played a minor role in the interaction between bxyWP and aflibercept. Besides, the stronger interaction between the penetratin derivatives and aflibercept promoted more efficient binding of peptide with aflibercept in the mixed solutions (Figure 1I). For the complex containing 30 mg mL^−1^ aflibercept, more than 99.8% of bxyWP was bound to aflibercept when the concentration of bxyWP was lower than 7.6 mg mL^−1^ (Figure S2, Supporting Information). We also observed larger complex particle size as the concentration of bxyWP in the AFL/bxyWP complex increased (Figure 1J; Figure S3, Supporting Information). Moreover, as shown in Figure S4A (Supporting Information), bxyWP could also interact with bevacizumab and ranibizumab, with K_d_ values of 5.95 ± 0.16 and 149.90 ± 27.67 µm, respectively. Due to these interactions, bxyWP also significantly enhanced the cellular uptake of  bevacizumab and ranibizumab in ARPE‐19 cells (Figure S4B, Supporting Information). These demonstrate the generalizability of our non‐covalent binding strategy and reproducibility of the interaction between the peptide bxyWP and positively charged proteins.

### 2.2 Reversible Binding Between Aflibercept and bxyWP is Indispensable for Improved Cellular Uptake

**Figure 2:**
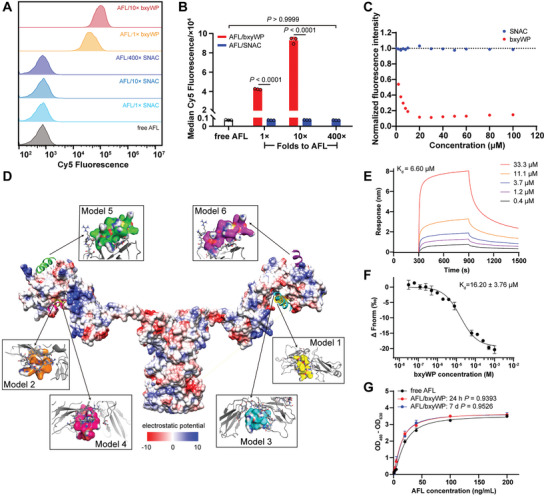
Binding pattern of bxyWP to aflibercept. A) Flow cytometry histograms and B) the median fluorescence intensity of ARPE‐19 cells. The cells were incubated with Cy5‐labelled free aflibercept and various complexes for 2 h at 37 °C (final concentration of aflibercept = 1 µm, *n* = 3). C) Binding curves of SNAC and bxyWP when incubated with aflibercept, evaluated by equilibrium fluorescence quenching assay at 25 °C in 1× PBS (*n* = 4). D) Binding sites of bxyWP on aflibercept were simulated by the Cluspro 2.0 server. Aflibercept was displayed in electrostatic surface potentials colored red (‐) and blue (+). bxyWP was displayed as cartoon in various colors indicating its potential binding sites on aflibercept. The interaction residues are shown as sticks in the zoom‐in sub‐images. E) Determination of the affinity between bxyWP and aflibercept using bio‐layer interferometry (BLI). F) Determination of the affinity between bxyWP and aflibercept using microscale thermophoresis (MST) (*n* = 3). G) ELISA‐based binding analysis showing that bxyWP did not interfere with the binding of aflibercept and VEGF_165_ (*n* = 3). Data are presented as means ± SD. Statistical analysis was performed using one‐way ANOVA with multiple comparisons corrected by Sidak's test in (B) or by Dunnett's test compared with the free aflibercept group in (G).

Sodium *N*‐[8‐(2‐hydroxybenzoyl) aminocaprylate] (SNAC) is a well‐characterized transcellular penetration enhancer that is co‐formulated with semaglutide (Rybelsus). It could promote oral absorption of semaglutide by increasing cellular permeability.^[^ ^25^ ^]^ Researches have demonstrated that due to the aromatic structure, SNAC could form hydrophobic interactions with several macromolecules, thereby improving their cellular uptake, much like the way that bxyWP affected on AFL.^[^ ^26^, ^27^ ^]^ This motivate us to explore whether SNAC could perform similarly on AFL to enhance absorption by forming a non‐covalent complex. The cellular uptake of aflibercept in ARPE‐19 cells revealed that SNAC failed to promote intracellular delivery even at a molar concentration 400‐fold that of aflibercept (**Figure** **2**A,B; Figure S5, Supporting Information). When the molar concentration of bxyWP or SNAC was ten‐fold that of aflibercept, the median fluorescence intensity of AFL/bxyWP complex treated cells was 127‐fold higher than that of AFL/SNAC treated cells. These results may be attributed to the minimal interaction between SNAC and aflibercept (Figure 2C), further emphasizing the necessity of bxyWP binding with aflibercept for promoting aflibercept uptake.

The binding affinity was further investigated using the ClusPo protein‐protein docking webserver. The molecular mechanics/generalized Born surface area free energy analysis was performed to calculate the binding free energy of the predicted peptide‐protein complexes. This analysis considered the van der Waals, electrostatic, hydrophobic and solvation interactions between bxyWP and aflibercept. The interacting residues between them were identified in the chosen complex structures with lower free energy (Figure 2D). Two binding pockets for bxyWP were observed on the surface of aflibercept R1D2 domain. The first binding pocket was a hydrophobic cavity, which was formed by three loops in models 1, 2, 3, and 4 for bxyWP binding. The three loops were loop 1 containing residues Leu46‐Asp47‐Thr48‐Leu49, loop 2 containing residues Asn91‐Try92‐Leu93‐Thr94‐His95‐Arg96, and loop 3 containing residues Val132‐Gly133‐Ile134‐Asp135. The second binding pocket was formed by a loop containing residues Asp52‐Gly53‐Lys54‐Arg55‐Ile56‐Ile57‐Trp58‐Asp59 and a beta‐sheet containing residues Phe64‐Ile65‐Ile66‐Ser67 in models 5 and 6. In model 1, Glu80 residue formed a salt bridge with R1 residue from bxyWP, and Leu46 residue formed hydrophobic stacks with W2 from bxyWP, enhancing the binding affinity. However, in model 3, these interactions were replaced by a salt bridge between Glu16 and K4 (bxyWP) and a hydrophobic interaction between Ile17 and I3 (bxyWP). The dissociation constants between bxyWP and aflibercept detected by bio‐layer interferometry (BLI) and microscale thermophoresis (MST) assays were 6.60 and 16.20 ± 3.76 µm (Figure 2E,F), respectively, which were close to the value determined using equilibrium fluorescence quenching assay. Besides, according to the binding curves evaluated by ELISA (Figure 2G), bxyWP did not affect the binding of aflibercept to its target VEGF_165_, and the binding activity of aflibercept remained stable over 7 days.

### 2.3 Aflibercept and bxyWP are Simultaneously Internalized without Being Interrupted by Endogenous Proteins

**Figure 3:**
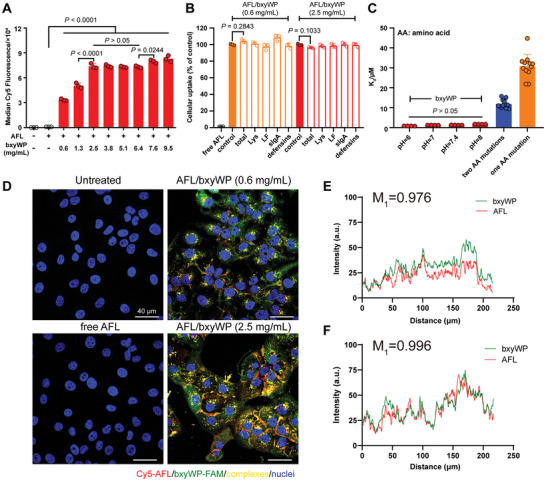
Cellular uptake behavior of aflibercept mediated by bxyWP. A) Flow cytometry analysis of the median fluorescence intensity in ARPE‐19 cells. The AFL/bxyWP complexes were pre‐formed by incubating 30 mg mL^−1^ Cy5‐labelled aflibercept with various concentrations of bxyWP, and then were diluted to contain 1 µm aflibercept. The complexes were incubated with ARPE‐19 cells for 2 h at 37 °C (*n* = 3). B) Major endogenous proteins in tears did not interfere with bxyWP‐mediated aflibercept entry into HCEC cells (*n* = 3). The internalization of Cy5‐AFL in the AFL/bxyWP (0.6 mg mL^−1^) and AFL/bxyWP (2.5 mg mL^−1^) groups without endogenous proteins was set as the control (100%). C) Determination of K_d_ for AFL/bxyWP complex using equilibrium fluorescence quenching assay at 25 °C in phosphate buffers at gradient pH values (*n* = 4 for left four columns, *n* = 12 for right two columns). (D) Merged confocal images of FAM‐labelled bxyWP (green) and Cy5‐labelled aflibercept (red) in HCEC cells. The AFL/bxyWP complexes were pre‐formed by incubating 30 mg mL^−1^ aflibercept with 0.6 mg mL^−1^ or 2.5 mg mL^−1^ bxyWP, and then were diluted to contain 1 µm aflibercept. The complexes were incubated with cells for 2 h at 37 °C before fixation. Nuclei were stained with DAPI (blue). Scale bar, 40 µm. E,F) Corresponding colocalization analysis and M_1_ coefficients (fraction of red fluorescence overlapping with green fluorescence) calculated by ImageJ for incubation with AFL/bxyWP complexes containing 0.6 and 2.5 mg mL^−1^ bxyWP, respectively. Data are presented as means ± SD. Statistical analysis was performed using one‐way ANOVA with multiple comparisons corrected by Tukey's test in (A, C) or by Dunnett's test in (B).

The cellular uptake of Cy5‐labelled aflibercept was quantitatively examined after being mixed with bxyWP at various concentrations. As shown in **Figure** **3**A, the median fluorescence intensity of ARPE‐19 cells treated with AFL/bxyWP complexes increased with the concentration of bxyWP in the complex until reaching a plateau at 2.5 mg mL^−1^. Therefore, we chose the combination of 30 mg mL^−1^ AFL and 2.5 mg mL^−1^ bxyWP for further evaluations and designated this formulation as the complex unless otherwise noted. Despite the presence of endogenous proteins from tears in the incubation medium, including lysozyme (Lys), lactoferrin (LF), secretory immunoglobulin A (sIgA), and defensins, the cellular uptake of aflibercept mediated by bxyWP remained largely unaffected (Figure 3B). Additionally, the K_d_ values kept stable when pH varied from 6.0 to 8.0, which contrasted sharply with those induced by single or double amino acid mutations in the peptide sequence, implying potential changes in ambient pH during the complex passage through ocular barriers did not impact the interaction between bxyWP and aflibercept (Figure 3C). To further validate simultaneous cellular uptake of bxyWP and aflibercept, the colocalization of fluorescein amidite (FAM)‐labelled bxyWP and Cy5‐labelled aflibercept were demonstrated in Figure 3D. AFL and bxyWP exhibited high colocalization, with the M_1_ colocalization coefficients increased from 0.976 to 0.996 as the concentration of bxyWP quadrupled (Figure 3E,F).

### 2.4 AFL/bxyWP Complex Traverses Cellular Barriers via Transcellular Pathway

**Figure 4:**
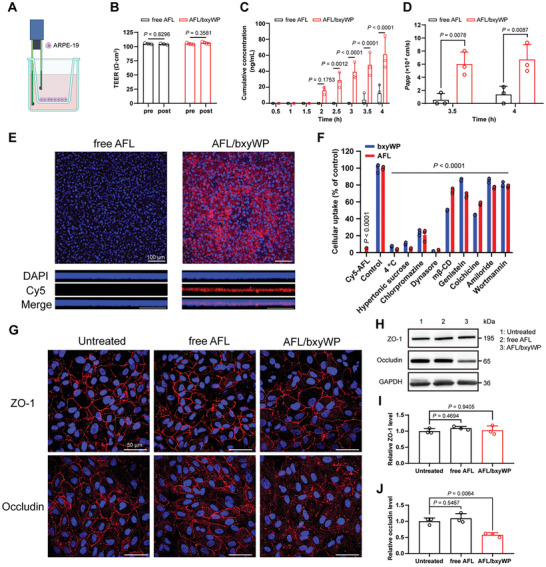
Mechanisms of AFL/bxyWP complex across cellular barrier. A) Schematic diagram of the in vitro RPE barrier model. B) Measurement of transepithelial electrical resistance (TEER) before and after incubation with complexes (*n* = 3). C) Cumulative concentration of transported aflibercept in the acceptor sides with free aflibercept or AFL/bxyWP complex in the donor sides (*n* = 3). D) Apparent permeability coefficient (*P*_app_, cm ^−1^s) of free aflibercept or AFL/bxyWP complex across the monolayer at 3.5 and 4 h (*n* = 3). E) Cross and vertical sections of confocal images of ARPE‐19 monolayer after incubation with Cy5‐labelled free aflibercept or AFL/bxyWP complex for 6 h. Nuclei were stained with DAPI (blue), red represented Cy5‐labelled aflibercept. Scale bar, 100 µm. F) Effects of temperature and endocytosis inhibitors on cellular uptake of AFL/bxyWP complex in ARPE‐19 cells (*n* = 3). FAM‐labelled bxyWP or Cy5‐labelled aflibercept internalization under uninhibited condition was set as control. G) Immunofluorescence images (scale bar, 50 µm) and (H) Western blotting images indicating the expression of tight junction‐related proteins, including ZO‐1 and occludin, in ARPE‐19 monolayer after incubation with free aflibercept or AFL/bxyWP complex for 2 h. Results of parallel experiments are provided in Figure S7 (Supporting Information). I,J) Statistical analysis of ZO‐1 and occludin in ARPE‐19 monolayer according to the Western blotting images (*n* = 3). Data are presented as means ± SD. Statistical analysis was performed using two‐way ANOVA with multiple comparisons corrected by Sidak's test in (B–D) and using one‐way ANOVA with multiple comparisons corrected by Dunnett's test compared with the control or untreated group in (F), (I), and (J).

An in vitro ARPE‐19 cell monolayer model (**Figure** **4**A) was established to investigate the transfer efficiency of the AFL/bxyWP complex across the retinal pigment epithelium (RPE) barrier.^[^ ^28^, ^29^ ^]^ After incubation with 1 µm aflibercept for 4 h, the monolayer remained intact (Figure 4B). In the initial 3 h, there was no free aflibercept traversing the in vitro RPE barrier. In contrast, the AFL/bxyWP complex showed more rapid and higher permeation (Figure 4C). The calculated apparent permeability coefficient (*P*_app_) of AFL/bxyWP was 10.9‐fold higher at 3.5 h and 4.9‐fold higher at 4 h compared to the free aflibercept, respectively (Figure 4D). Significantly brighter red fluorescence in cross section and deeper infiltration in vertical section (Figure 4E) further illustrated bxyWP was capable of transporting aflibercept to the retina.

The cellular uptake of FAM‐labelled bxyWP and Cy5‐labelled aflibercept were simultaneously and significantly inhibited by low temperature (4 °C), hypertonic sucrose, chlorpromazine, and dynasore, suggesting that the AFL/bxyWP complex was internalized via energy‐dependent cellular uptake and clathrin‐mediated endocytosis (Figure 4F; Figure S6, Supporting Information). When the cellular uptake of bxyWP was inhibited, the uptake of AFL almost declined simultaneously, demonstrating that the internalization of bxyWP and AFL was a synchronized process, consistent with the results concluded from Figure 3D. Effective cellular uptake of bxyWP was a necessity for enhancing AFL absorption. Moreover, bxyWP promoted aflibercept penetration across the RPE barrier by slightly down‐regulating the level of tight junction protein occludin but did not completely disrupt tight junctions, as evidenced by the maintained ZO‐1 level (Figure 4G–J; Figure S7, Supporting Information) and transepithelial electrical resistance (TEER) (Figure 4B; Figure S8, Supporting Information). After exposed to the complex, the expression level of occludin returned to normal in 24 h (Figure S9, Supporting Information). In contrast, the AFL/bxyWP complex of the same concentration did not influence the tight junction proteins in HCEC monolayer (Figure S10, Supporting Information). In summary, the mechanism by which AFL/bxyWP complex traversed the cellular barriers of the posterior ocular segment was dominated by the transcellular pathways, with a supplementary contribution from the paracellular pathway.

### 2.5 AFL/bxyWP Eye Drops Improve Ocular Retention and Penetration to Posterior Segment

**Figure 5:**
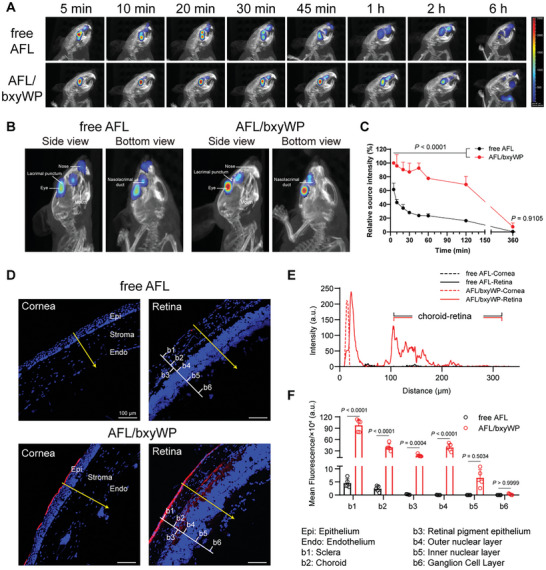
Retention and penetration of AFL/bxyWP complex in rodent eyes. A) IVIS Spectrum CT images of Cy5‐labelled aflibercept in the eyes after topical instillation of free aflibercept or AFL/bxyWP complex (30 mg mL^−1^ aflibercept, 3 µL per eye) at different time points. Results of parallel experiments are provided in Figure S11 (Supporting Information). B) Side and bottom views of the ocular and nasal regions at 45 min after topical instillation of free aflibercept or AFL/bxyWP complex. C) Elimination curves of aflibercept from the eye (*n* = 3). The total values of source intensity at 5 min for free aflibercept or AFL/bxyWP complex were set as 100%, respectively. D) Confocal images of intraocular penetration of topically instilled free aflibercept and AFL/bxyWP complex. The eyes were collected 1 hour after the last administration to prepare DAPI‐stained frozen sections (scale bar, 100 µm). E) The fluorescence intensity of aflibercept from corneal epithelium to endothelium and from sclera to inner retina was plotted using ImageJ (*n* = 5). F) Quantitative analysis of the fluorescence intensity of aflibercept at different tissues in the posterior segment of the eye. Data are presented as means ± SD. Statistical analysis was performed using two‐way ANOVA with multiple comparisons corrected by Sidak's test in (C) and (F).

To estimate ocular retention and distribution of AFL/bxyWP complex after topical instillation, aflibercept was labelled with Cy5 and administered in rodents. Due to the addition of the positively charged bxyWP, the AFL/bxyWP complex possessed a favorable ability to bind to the negatively charged ocular surface, thus substantially prolonging the retention time of aflibercept in the eye (**Figure** **5**A; Figure S11, Supporting Information). As shown in the merged 3D imaging of CT and fluorescent signal, free aflibercept was quickly eliminated into the nasal cavity through the nasolacrimal duct (Figure 5B). In contrast, most of the aflibercept in the complex remained in the eye and spread all over the eyeball. The residual amounts of aflibercept were 23.84 ± 0.53% versus 92.54 ± 7.44% at 45 min after topical instillation of free aflibercept or AFL/bxyWP complex, respectively. The elimination rate of aflibercept in the complex was evidently slower compared to the free aflibercept within 2 h after eye drops administration, as revealed by the significantly stronger fluorescent intensity in the eye (Figure 5C). The area under curve from 0 to 2 h calculated by the trapezoidal method for the AFL/bxyWP group was 3.2 times higher than that of the free AFL group. With the aid of bxyWP, the instilled aflibercept was able to penetrate the superficial corneal epithelium and, importantly, reach the posterior segment of the eye, distributing in all layers of sclera, choroid, and retina (Figure 5D–F).

### 2.6 AFL/bxyWP Eye Drops are Absorbed via Conjunctival‐Scleral Pathway

**Figure 6:**
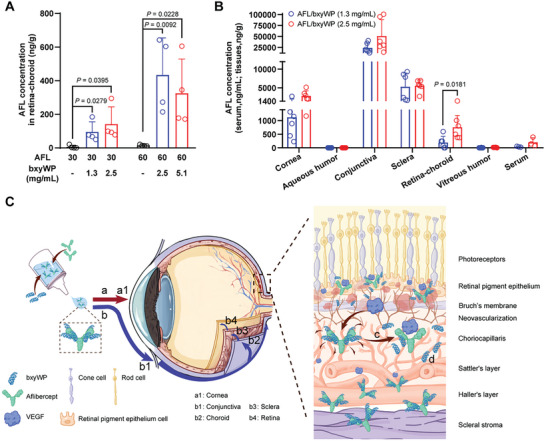
Distribution of aflibercept in rabbit eye and serum after topical instillation of AFL/bxyWP complexes. A) Concentration of aflibercept in retina‐choroid 1 hour after a single eye drops administration of free aflibercept or AFL/bxyWP complexes (50 µL per drop, two drops each administration, *n* = 4). The AFL/bxyWP complexes contained 30 mg mL^−1^ aflibercept with 1.3 or 2.5 mg mL^−1^ bxyWP, or 60 mg mL^−1^ aflibercept with 2.5 or 5.1 mg mL^−1^ bxyWP. B) Concentration of aflibercept in ocular tissues and serum 1 hour after the last dose of AFL/bxyWP complexes (containing 30 mg mL^−1^ aflibercept and either 1.3 or 2.5 mg mL^−1^ bxyWP) given 3 times a day at a 4 h interval via topical instillation. The volume of administration was 30 µL per drop for AFL/bxyWP (1.3 mg mL^−1^) complex and 50 µL per drop for AFL/bxyWP (2.5 mg mL^−1^) complex (two drops each administration, *n* = 6 for ocular tissues and *n* = 3 for serum). Data are presented as means ± SD. Statistical analysis was performed using two‐tailed unpaired *t*‐test. C) Schematic diagram of the ocular absorption pathway of the AFL/bxyWP complex: a, corneal pathway; b, conjunctival‐scleral pathway; c, binding of aflibercept to VEGF; d, released bxyWP would be degraded in serum or ocular tissues.

Due to the presence of ocular barriers, the delivery of hydrophilic biomacromolecules to the posterior segment of the eye following topical administration is extremely difficult. However, aflibercept concentration in the retina‐choroid of rabbits substantially increased with addition of bxyWP in the eye drops (**Figure** **6**A). When the concentration of bxyWP reached 2.5 mg mL^−1^ in the 30 mg mL^−1^ aflibercept formulation, the aflibercept concentration in retina‐choroid was 141.22 ± 102.68 ng g^−1^ after single dose, which was much higher than the median effective concentration (EC_50_) of aflibercept (39.1 ng mL^−1^).^[^ ^30^ ^]^ When fixing the ratio of bxyWP to aflibercept and increasing the concentration of aflibercept in the eye drops from 30 to 60 mg mL^−1^, even higher aflibercept concentrations ranging from 324.97 ± 203.69 to 433.29 ± 221.30 ng g^−1^ were achieved in the retina‐choroid, suggesting that the transport of the complex to the posterior segment was predominantly driven by passive diffusion. Furthermore, the absorption via conjunctiva‐sclera route was quantitatively demonstrated by undetectable aflibercept in aqueous humor and the gradual decrease in aflibercept concentrations from conjunctiva, sclera, retina‐choroid to vitreous humor after administration of AFL/bxyWP eye drops (Figure 6B,C). The cumulative concentrations of aflibercept in the retina‐choroid were 290.40 ± 222.00 and 756.67 ± 432.47 ng g^−1^ respectively, after applying a daily dose of AFL/bxyWP complexes containing either 1.3 mg mL^−1^ bxyWP with a total of 5.4 mg aflibercept or 2.5 mg mL^−1^ bxyWP with a total of 9 mg aflibercept. In contrast, for free aflibercept eye drops, the aflibercept concentration in the retina‐choroid remained at a low level (16.57 ± 23.87 ng g^−1^), even with a total of 9 mg aflibercept administered (Figure S12, Supporting Information). Under the higher daily dose, the aflibercept concentration in the serum was 202.17 ± 169.73 ng mL^−1^, which was much lower than the peak plasma concentration (1600 ng mL^−1^) of rabbits subjected to intravitreal injection of 0.5 mg aflibercept.^[^ ^31^ ^]^ According to these results, the transport of aflibercept into the eye mediated by bxyWP was dose‐ and formulation‐dependent with low systemic exposure.

### 2.7 AFL/bxyWP Eye Drops Efficiently Reverse CNV in Cynomolgus Monkeys

**Figure 7:**
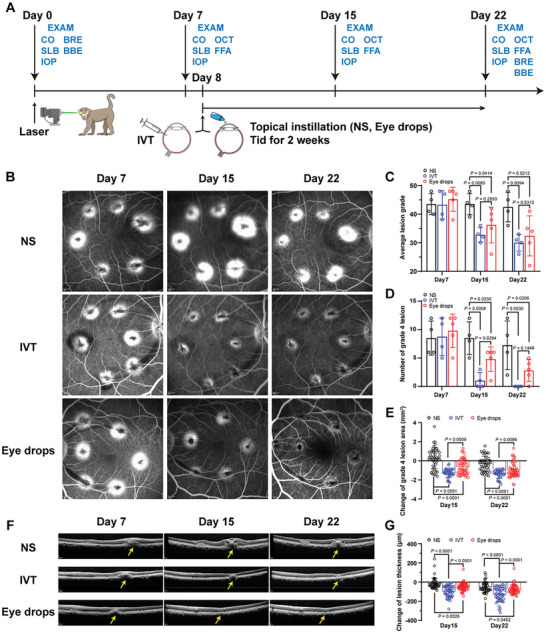
Efficacy of AFL/bxyWP eye drops on laser‐induced CNV lesions in cynomolgus monkeys. A) Time schedule of CNV‐bearing monkeys receiving treatment and evaluation. Monkeys (*n* = 13) were randomly divided into three groups and treated with a single intravitreal injection of 40 mg mL^−1^ aflibercept (positive control group, IVT, 50 µL per eye), topical instillation of normal saline (negative control group, NS) or AFL/bxyWP complex (experimental group, Eye drops, 30 mg mL^−1^ aflibercept, 50 µL per drop, two drops each administration) three times daily for 2 weeks (*n* = 4 for NS and IVT groups, *n* = 5 for Eye drops group). B) Representative fundus fluorescein angiography showing CNV leakage on day 7, 15, and 22 after modeling. The monkeys received intravenously injected fluorescein sodium (10 mg kg^−1^) before detection. C) Average lesion grade from day 7 to day 22 after modeling, calculated as the sum of spot scores for both eyes of each animal. D) Number of grade 4 lesion in both eyes of each animal from day 7 to day 22 after modeling. E) Change of grade 4 lesion area, calculated as changes in the area of each grade 4 spot (*n* = 45 for NS group, *n* = 35 for IVT group, and *n* = 50 for Eye drops group). F) Representative optical coherence tomography images of retina obtained on day 7, 15, and 22 after modeling. Lesions are indicated by yellow arrows. G) Change of lesion thickness on day 15 and 22 relative to day 7 after modeling, calculated as changes in the thickness of each spot (*n* = 48 for NS group, *n* = 47 for IVT group, *n* = 59 for Eye drops group). Data are presented as means ± SD. Statistical analysis was performed using Levene's test and one‐way ANOVA with LSD post‐hoc test for multiple comparisons. Notes: CO, clinical observation; SLB, slit lamp biomicroscopy; IOP, intraocular pressure; FFA, fundus fluorescein angiography; OCT, optical coherence tomography; BRE, blood routine examination; BBE, blood biochemistry examination; IVT, intravitreal injection; NS, normal saline.

The in vivo evaluation of AFL/bxyWP complex for the treatment of nAMD was performed using the laser‐induced CNV model on cynomolgus monkeys (**Figure** **7**A). Representative fluorescein angiography images taken on day 7, 15, and 22 after the laser exposure are shown in Figure 7B for the negative control group (NS, receiving normal saline eye drops), the positive control group (IVT, receiving a single intravitreal injection of aflibercept on day 8), and the experimental group (Eye drops, receiving instilled AFL/bxyWP complex, three times a day from day 8 for 2 weeks). All these groups developed comparable CNV lesions on day 7 with high‐grade leakage on fluorescein angiograms. The average lesion grade reduced significantly in the IVT and Eye drops groups by day 15 and remained lower than the NS group until the endpoint of the study, with no statistical difference between the IVT and Eye drops groups (Figure 7C). The average lesion grade of the NS group decreased by only 1 point from day 7 to day 22, while the IVT and Eye drops groups decreased by 13.2 and 12.8 points, respectively. Likewise, the number of grade 4 CNV lesions was significantly fewer in the IVT and Eye drops groups by day 15 as compared to the NS group, with no significant difference between the IVT and Eye drops groups by the endpoint of the study (Figure 7D). The grade 4 lesion area was significantly reduced in both the IVT and Eye drops groups as compared to the NS group on day 15 and day 22. However, the reduction in lesion area was significantly more in the IVT group as compared to Eye drops group at both the day 15 and day 22 time points (Figure 7E).

Representative examples of the CNV lesions observed on optical coherence tomography (OCT) and their response to treatment are shown in Figure 7F. The CNV lesion thickness was significantly reduced in both the IVT and Eye drops groups at day 15 and day 22 as compared to the NS group, although the IVT group recovered better than the Eye drops group during treatment (Figure 7G). These results demonstrate that the AFL/bxyWP eye drops were able to effectively reverse the CNV lesions in the cynomolgus monkey model, although the treatment effect in 2 weeks was slightly less pronounced compared to the intravitreal injection of aflibercept.

### 2.8 AFL/bxyWP Eye Drops are Safe to Cynomolgus Monkeys

**Figure 8:**
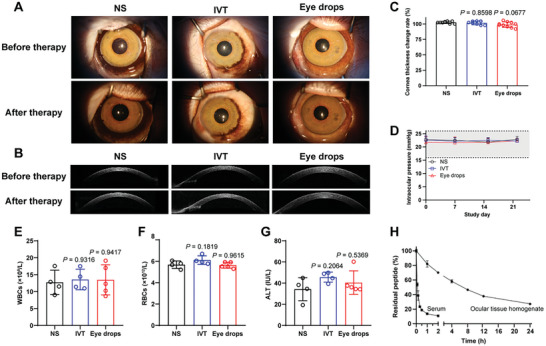
Safety assessment of AFL/bxyWP eye drops. A) Representative images of slit‐lamp microscopy and B) OCT of cornea treated with normal saline (negative control group, NS), AFL/bxyWP eye drops (experimental group, Eye drops) and a single intravitreal injection of aflibercept (positive control group, IVT). C) Statistical analysis of cornea thickness according to the OCT images and D) intraocular pressure of monkeys treated with normal saline, AFL/bxyWP eye drops and a single intravitreal injection of aflibercept (*n* = 8 for NS and IVT groups, *n* = 10 for Eye drops group). E–G), Blood routine examination and blood biochemistry examination of monkeys after therapy (*n* = 4 for NS and IVT groups, *n* = 5 for Eye drops group). Statistical analysis was performed using one‐way ANOVA with multiple comparisons corrected by Dunnett's test compared with the NS group. H) Degradation of bxyWP in rabbit serum and ocular tissue homogenates at 37 °C determined by HPLC (*n* = 3). Data are presented as means ± SD. Notes: WBC, white blood cell; RBC, red blood cell; ALT, alanine aminotransferase; NS, normal saline; IVT, intravitreal injection.

During the safety evaluation, general clinical observations and ophthalmic examinations showed no significant abnormalities (**Figure** **8**A; Figure S13, Supporting Information), indicating that the AFL/bxyWP eye drops did not cause obvious eye irritation, inflammation or toxicity. No significant changes in corneal thickness or intraocular pressure were observed before and after the therapy (Figure 8B–D). Hematological parameters of AFL/bxyWP‐treated monkeys were similar to those of normal saline‐treated monkeys, indicating no signs of inflammation, immune responses (Figure 8E; Figure S14A–C, Supporting Information), or other systemic side effects (Figure 8F,G; Figure S14D–K, Supporting Information). Based on the ex vivo degradation profiles, the calculated half‐life of bxyWP in rabbit serum was 6.25 min at 37 °C (Figure 8H), implying that the peptide would be rapidly degraded by enzymes in the blood if it entered systemic circulation through the conjunctiva or nasolacrimal duct, which was conducive to the biological safety. In comparison, the degradation rate of bxyWP in ocular tissue homogenates was relatively slower, with a half‐life of 6.82 h. Nevertheless, the actual degradation of the peptide in the eye will accelerate due to the constant reduction of enzyme activity in vitro.

## 3 Discussion

Intravitreal injections of anti‐VEGF agents have to date been the first‐line therapeutic regimen for neovascular retinal diseases, including nAMD. However, these invasive injection procedures are associated with complications including endophthalmitis, elevated intraocular pressure, cataracts, and even retinal detachments.^[^ ^32^ ^]^ Moreover, the considerable treatment burden and, in certain countries, the cost of frequent injections lead many patients to abandon their treatment regimens early. To improve the safety and compliance of anti‐VEGF treatment, a less invasive and more convenient ocular drug delivery system is urgently needed. Previous studies on CPPs promoting ocular absorption of functional proteins via eye drops mainly employed strategies of covalent conjugation or fusion proteins, for example, PEP‐1‐FK506BP,^[^ ^33^ ^]^ TAT‐aFGF‐His,^[^ ^34^ ^]^ and TAT PTD‐endostatin.^[^ ^35^ ^]^ In contrast, our non‐covalent delivery strategy of incorporating CPPs as absorption enhancers into protein formulations has significant advantages: i) no need to change the primary structure of the biomacromolecule, with minimal impact on protein conformation and biological activity; ii) the ratio of CPP to protein can be adjusted, allowing for flexible and convenient formulation designs; and iii) the preparation process only requires simple mixing, which is easy to implement and scale up.

It was previously reported that polyarginine could facilitate bevacizumab penetration into *ex vivo* porcine eyes.^[^ ^36^ ^]^ However, the delivery mechanism was not clearly elucidated. Our study found that low concentration of polyarginine R_8_ failed to promote cellular uptake of aflibercept because they are both positively charged, and their interaction was therefore negligible. Similarly, wild‐type penetratin, whose hydrophilicity is second only to R_8_, showed the same result. Although previous reports suggest that positively charged CPPs could form non‐covalent complexes with anti‐VEGF agents via electrostatic interaction,^[^ ^37^, ^38^ ^]^ our results revealed that electrostatic interaction and hydrogen bonding played only minor roles in the binding between bxyWP and aflibercept, as demonstrated by the minimal changes in fluorescence intensity and K_d_ value upon the addition of higher salt concentration and urea, which respectively affected electrostatic interaction and hydrogen bonding. The hydrophobic interaction played a dominant role, as evidenced by the significantly elevated binding affinity of aflibercept with penetratin derivatives that had greater hydrophobicity. By introducing no more than three hydrophobic tryptophan, the derived peptide bxyWP maintained good solubility (higher than 110 mg mL^−1^ in normal saline), while ensuring that the hydrophobic interaction between bxyWP and aflibercept remained moderate. This finding was confirmed using computer simulations that showed models with hydrophobicity‐favored interactions exhibited lower free energy and therefore more stable and physiologically relevant complex structures. A hydrophobic cavity was observed on the surface of aflibercept R1D2 domain, serving as the binding pocket for bxyWP. The K_d_ value between aflibercept and bxyWP obtained using BLI and MST assays approximated the result obtained by the equilibrium fluorescence quenching assay, further confirming our hypothesis. More importantly, the nearly hundredfold increase in the cellular uptake of aflibercept strongly implied the powerful transmembrane transport capacity brought by this interaction. Furthermore, the relatively strong hydrophobic interaction enabled bxyWP to effectively mediate aflibercept across the ocular absorption barriers, representing a promising non‐covalent delivery strategy for the treatment of neovascular eye diseases.

These observations were reinforced when compared to SNAC, a well‐known penetration enhancer that could improve the absorption of semaglutide in the stomach. In addition to protecting semaglutide against enzymatic degradation via local buffering action and promoting monomerization,^[^ ^25^ ^]^ some studies have reported that SNAC and peptides could form non‐covalent complexes to enhance absorption.^[^ ^26^, ^27^ ^]^ Herein, we compared the ability of SNAC and bxyWP to promote the cellular uptake of aflibercept and found that bxyWP outperformed SNAC. Buckley et al. found that a sufficiently high concentration of SNAC was necessary to promote the absorption‐enhancing effects, so we added 400‐fold SNAC compared to aflibercept in the formulation. However, even at such a high concentration, SNAC failed to facilitate the absorption of aflibercept. In contrast, at a much lower fold concentration, bxyWP was able to promote cellular uptake of aflibercept. The absorption‐enhancing effects of SNAC were observed to be size‐dependent and compound specific. When the molecular weight of the cargo exceeds 4 kDa, the effect diminishes.^[^ ^25^ ^]^ Aflibercept has a molecular weight of 115 kDa, much larger than this critical value. Thus, SNAC was too small to interact with aflibercept and in turn, failed to promote absorption. Based on these findings, we strongly conclude that the hydrophobic interaction is the critical factor enabling bxyWP to facilitate aflibercept's passage through the absorption barrier. With this hydrophobic interaction, AFL/bxyWP complex can penetrate into the eye without being dissociated by endogenous proteins in the tear film or being affected by altered ambient pH, until reaching the CNV lesion.

Absorption enhancers exert their effects in various ways. Chelating agents like ethylenediamine‐N,N,N’,N’‐tetraacetic acid enhance drug penetration via the disruption of tight junctions. Non‐ionic surfactants with lipophilic and hydrophilic moieties, such as Tween and Span, act by perturbing phospholipid acyl chains of cell membranes.^[^ ^39^ ^]^ The function mode of bxyWP to mediate the delivery of aflibercept was predominantly through energy‐dependent and clathrin‐mediated endocytosis, supplemented by caveolae‐mediated endocytosis and macropinocytosis. Following endocytosis, bxyWP may escape from the endosome into the cytosol through the so‐called vesicle budding‐and‐collapse mechanism,^[^ ^40^, ^41^ ^]^ enabling further translocation of aflibercept. Nevertheless, cellular uptake mechanisms are associated with various factors, including CPP concentration, cell type, membrane potential and even the pH condition.^[^ ^42^ ^]^ The conclusions we have obtained so far are limited to the specific concentration and cell type studied for bxyWP and its cargo, aflibercept. Considering that posterior segment penetration of AFL/bxyWP complex involves various tissues and cell types, and the concentration of bxyWP also differs across the layers, the precise mechanisms underlying layer‐by‐layer penetration remain to be fully elucidated. We hypothesize that, in addition to the above‐mentioned endocytosis, mechanisms such as direct translocation are likely involved simultaneously. It has been reported that at higher CPP concentrations, direct translocation across membranes becomes more probable.^[^ ^43^ ^]^ For the bxyWP in our study, it contains three arginine residues, whose guanidinium groups can mediate direct translocation through hydrogen bonding interactions with phosphate groups in the cell membrane. Besides, high content of α‐helix in bxyWP^[^ ^24^ ^]^ and hydrophobic tryptophan residues can also facilitate pore formation.^[^ ^44^, ^45^ ^]^ Results from Figure S6 (Supporting Information) partially indicate that at a higher concentration of bxyWP, aflibercept could be internalized into ARPE‐19 cells via an energy‐independent pathway, with ≈80% cellular uptake of control at low temperature. Additionally, the transport of AFL/bxyWP complex was mediated by a minor down‐regulation of occludin, a tight junction protein in the RPE, which did not disrupt the barrier function. Interestingly, unlike in ARPE‐19 cells, the levels of tight junction proteins, occludin and ZO‐1, remained almost unchanged in HCEC monolayer after incubation for 2 h with the AFL/bxyWP complex (Figure S10, Supporting Information). This phenomenon is consistent with the more dense and impermeable nature of the corneal epithelial layer.

The human tear film has a turnover rate of 14.9 ± 5.6% per minute,^[^ ^46^ ^]^ leading to a quick clearance of topical drugs from the ocular surface into the nasolacrimal duct. This short retention time is one of the major reasons for low ocular bioavailability of topically administered drugs. Our results show that the positively charged bxyWP could prolong the retention time of aflibercept on the ocular surface greater than 2 h, postpone nasolacrimal drainage, and provide more time for bxyWP to facilitate the diffusion of aflibercept to the retina. Considering the impermeable property of the cornea and its considerable distance to the fundus, the AFL/bxyWP complex was verified to enter the eye through the conjunctiva‐sclera‐choroid‐retina pathway (Figure 6C), aligning with the penetration pathways of biomacromolecules found in previous studies.^[^ ^47^, ^48^ ^]^ When the rabbits received AFL/bxyWP eye drops three times daily, an accumulated concentration of aflibercept was achieved in the retina‐choroid, which was 45.7 times higher than that of the free aflibercept treated group, far exceeding the EC_50_ of aflibercept. The reported K_d_ value between aflibercept and VEGF_165_ was 0.49 pm.^[^ ^49^ ^]^ After reaching the CNV lesion, the higher affinity between aflibercept and overexpressed VEGF would displace bxyWP in the non‐covalent complex, allowing aflibercept to neutralize VEGF, while the free peptide would be rapidly degraded by the protease in ocular tissues or blood circulation.

When applied in real clinical scenarios, physiological and pathological differences among patients may affect the ocular pharmacokinetics of AFL/bxyWP eye drops. AMD mainly affects the elderly. In general, there is a negative correlation between age and choroidal thickness.^[^ ^50^ ^]^ Nevertheless, Invernizzi et al. found that the eyes of patients with active CNV were usually experiencing an increase in choroidal thickness and choroidal vascularity index (CVI).^[^ ^51^ ^]^ Moreover, the thickness of choroid would decrease during the anti‐VEGF therapies.^[^ ^52^ ^]^ Thus, when the AFL/bxyWP complex reaches the choroid via conjunctival‐scleral route, individual differences in choroidal thickness and CVI may impact its absorption. To be more specific, thinner choroid and sparser blood flow would facilitate deeper penetration of the complex into the eye, and vice versa. Besides, as the vitreous ages, components that maintain its viscoelastic nature, such as collagen fibrils and hyaluronic acid, begin to degenerate, which leads to vitreous liquefaction.^[^ ^53^, ^54^ ^]^ Importantly, reduced steric hindrance and enhanced convective flow after vitreous liquefaction allow the biomolecules diffuse faster in the vitreous humor.^[^ ^55^ ^]^ Although the AFL/bxyWP complex does not diffuse from the vitreous humor to the retina, the impact of vitreous convection on the overall intraocular fluid dynamics cannot be neglected, which may affect the diffusion of the complex in the eye. According to the Pharmacology Review of Eylea (aflibercept, BLA#125 387) disclosed by the FDA, the half‐life (t_1/2_) of free aflibercept in rabbit eyes is ≈5 days, while the calculated t_1/2_ of aflibercept bound to VEGF is up to ≈130 days. This indicates that under the pathological state of nAMD, where VEGF is overexpressed in the lesions involving choroid and retina, once aflibercept reaches the lesions with the assistance of bxyWP, it will bind to VEGF and acquire an extended half‐life. This significantly prolongs the retention time of aflibercept within the eye, thereby maintaining its efficacy for a relative longer duration. This also means that the amount of VEGF in the lesions may be relevant to the t_1/2_ of total aflibercept. Other pathological changes relating to retinal disorders such as permeability of Bruch's membrane and endothelial monolayer, and dysfunction of the complement system^[^ ^56^ ^]^ vary from patient to patient and may also influence the absorption of AFL/bxyWP eye drops in clinical applications.

The successful delivery of AFL/bxyWP eye drops was subsequently demonstrated by the therapeutic outcomes in the cynomolgus monkey nAMD model, which revealed efficient suppression of laser‐induced CNV lesions within two weeks and without clinical signs of ocular inflammation or toxicity. This is, to the best of our knowledge, the first proof that topically administered protein eye drops are effective for treating a neovascular retinal disease in non‐human primates. These findings demonstrate a promising transition from intravitreal injections to eye drops administration of anti‐VEGF, which holds significant clinical implications for patient care. In addition to nAMD, the FDA has also approved four indications associated with ocular neovascularization, including macular edema following retinal vein occlusion, diabetic macular edema, diabetic retinopathy and retinopathy of prematurity. These conditions could all potentially benefit from AFL/bxyWP eye drops, bringing new hope for improved patient outcomes. So far, more than eight protein drugs have been approved to treat fundus diseases by intravitreal or systemic injection.^[^ ^57^ ^]^ The non‐covalent delivery strategy can also be applied to other therapeutic proteins such as bevacizumab, ranibizumab (Figure S4B, Supporting Information) and adalimumab (ADA, Figure S15, Supporting Information) according to the substantially improved cellular uptake mediated by bxyWP. This generalizability demonstrates its potential to revolutionize the protein and antibody treatment for ocular diseases, paving the way for more effective therapies that can significantly improve patient compliance.

## 4 Conclusion

In summary, our study demonstrates that a peptide‐based ocular absorption enhancer may advance the treatment of neovascular retinal diseases, revolutionizing the current clinical practice from intravitreal injections to noninvasive eye drops administration. The optimal peptide, bxyWP, can spontaneously bind to aflibercept via hydrophobic interaction, leading to a universal topical treatment option for potential retinal vascular diseases. Our results provide a proof of concept that AFL/bxyWP is a safe and effective eye drops formulation, which successfully treated the laser‐induced CNV monkey model. These promising results will now need validation in future clinical trials. This work also offers further insights into the design of peptide‐based absorption enhancers and lays the foundation for elucidating their mechanisms in mediating ocular delivery of protein drugs. Our strategy may serve as a promising technology platform for delivering other therapeutic proteins or antibodies into the eye, bringing hope to patients suffering from various ocular diseases.

## 5 Experimental Section

### Manufacturing of CPP

Cell‐penetrating peptides (CPPs) were synthesized in small batches in the laboratory via solid‐phase synthesis,^[^ ^58^ ^]^ among which bxyWP was manufactured in large batches by Shengnuo Biopharmacy (Chengdu, China).

### Preparation of Aflibercept and CPP Complexes

Aflibercept (AFL) was provided by Qilu Pharmaceutical and dissolved in 10 mmol L^−1^ sodium phosphate buffer containing 5% sucrose, 40 mmol L^−1^ sodium chloride, and 0.03% Tween 20. CPPs were dissolved in 0.9% sodium chloride. AFL and CPP solutions were mixed at 3:1 volume ratio and incubated for 24 h at 4 °C before usage.

### Fluorescence‐Based Equilibrium Binding Assay

To measure the dissociation constants (K_d_) for AFL/CPP complexes, equilibrium fluorescence quenching assays were conducted at 25 °C in 1× PBS according to previously reported protocols^[^ ^59^ ^]^ with minor modifications.

Aflibercept was labeled with fluorescein isothiocyanate (FITC) using Quick FITC Protein Labelling Kit according to the manufacturer's instructions (Meilun Biotechnology) to obtain FITC‐AFL. The exact excitation (Ex) and emission (Em) wavelengths of FITC‐AFL in 1× PBS at pH 7.2 were determined using Cary Eclipse Fluorescence Spectrophotometer (Agilent Technologies).

Fluorescence of FITC‐AFL was monitored as the concentration of CPPs in the mixed solution increased until saturation was achieved. Each well had a total volume of 200 µL with 200 nm FITC‐AFL and corresponding peptide concentrations. Stock solutions of 200 µm CPPs, including R_8_, PE, bWP, xWP, yWP, bxWP, byWP, xyWP, and bxyWP, were used to prepare successively diluted solutions from 100 to 1 µm (diluted in 1× PBS), which were then gently mixed with FITC‐AFL solution. Each plate also contained a positive control with only 200 nm FITC‐AFL in 1× PBS and a negative control with only 200 µL 1× PBS. Plates were incubated at room temperature in the dark for 30 min to achieve equilibrium prior to measurements. Then, the fluorescence intensity of each well was measured by Synergy H1 microplate reader (BioTek) with Gen5 3.05 software (Ex 496 nm/Em 522 nm) and normalized to the positive control after subtracting the negative control. The K_d_ values were calculated using GraphPad Prism 8 software according to the fractional saturation plots^[^ ^60^ ^]^ converted from the normalized binding curves.

Furthermore, the K_d_ for the AFL/bxyWP complex was determined using the same assay at 25 °C in 3× PBS, or in 1× PBS with the addition of 0.5 m urea, or in phosphate buffer at pH 6.0, 7.0, 7.4, and 8.0. Fluorescence intensity of FITC‐AFL was also measured in the existence of 1 to 100 µm SNAC at 25 °C in 1× PBS.

### Cell Culture

HCEC and human umbilical vein endothelial cells (HUVEC) were purchased from BeNa Culture Collection and Stem Cell Bank (Chinese Academy of Sciences) respectively, and were cultured in Dulbecco's modified eagle medium (DMEM, ThermoFisher Scientific) supplemented with 10% fetal bovine serum (FBS, ThermoFisher Scientific) and 100 U mL^−1^ penicillin‐streptomycin (ThermoFisher Scientific) at 37 °C in a 5% CO_2_ humidified atmosphere. ARPE‐19 cells were provided by Stem Cell Bank (Chinese Academy of Sciences) and were cultivated in DMEM/Nutrient Mixture F‐12 (DMEM/F12, ThermoFisher Scientific) supplemented with 10% FBS and 100 U mL^−1^ penicillin‐streptomycin at 37 °C in a 5% CO_2_ humidified atmosphere.

### Cellular Uptake and Endocytosis Mechanism

HCEC, ARPE‐19, or HUVEC cells were seeded on sterile 12‐well plates (Xinyou Biotechnology) at a density of 1.5 × 10^5^ cells per well and incubated in a humidified incubator (ThermoFisher Scientific) at 37 °C with 5% CO_2_ overnight. Aflibercept was labeled by reacting with 1.5‐fold molar concentration of Cy5‐N‐hydroxysuccihnimide (NHS) ester (Meilun Biotechnology) at 4 °C in dark overnight to obtain Cy5‐labelled aflibercept (Cy5‐AFL). FAM‐labelled bxyWP was synthesized by Quantai Biotechnology. Cy5‐AFL was separately mixed with peptides and SNAC on the basis of molar concentrations. To compare the cellular uptake enhanced by various peptides, equimolar concentration of Cy5‐AFL and peptides was used. To investigate the effect of SNAC on the cellular uptake of Cy5‐AFL, 10‐, and 400‐fold higher molar concentrations of SNAC were used additionally. For the formulation of AFL/bxyWP complexes, Cy5‐AFL at 30 mg mL^−1^ were mixed with 0.6, 1.3, 2.5, 3.8, 5.1, 6.4, 7.6, and 9.5 mg mL^−1^ bxyWP, respectively. These solutions were diluted to contain 1 µm Cy5‐AFL. All the complexes were incubated with HCEC or ARPE‐19 cells for 2 h at 37 °C, and flow cytometry was employed for detection (Ex 638 nm/Em 660 nm).

To determine the interference with cellular uptake by endogenous proteins in tears, the AFL/bxyWP complexes were pre‐formed by incubating 30 mg mL^−1^ Cy5‐AFL with 0.6 or 2.5 mg mL^−1^ bxyWP. Subsequently, these solutions were diluted to contain 1 µm Cy5‐AFL and incubated with HCEC cells for 2 h at 37 °C in a culture medium containing major endogenous proteins in tears, and flow cytometry was employed for detection as above. The concentrations of proteins were: 90 nm lysozyme (Beyotime), 10 nm lactoferrin (Macklin), 1.33 nM sIgA (Sigma‐Aldrich), and defensins including 0.16 pm human neutrophil peptide‐1 and 0.015 pm human beta‐defensin 2.^[^ ^61^, ^62^ ^]^ Cy5‐AFL internalization without the existence of endogenous proteins was set as a control (100%).

To identify the internalization mechanism, ARPE‐19 cells were pretreated at 4 °C or with specific inhibitors^[^ ^63^ ^]^ at 37 °C for 1 h, followed by incubation with 1 µm Cy5‐AFL/bxyWP‐FAM at 4 °C or in the presence of inhibitors at 37 °C for another 2 h, and flow cytometry was employed for detection (Ex 488 nm/Em 525 nm for bxyWP‐FAM and Ex 638 nm/Em 660 nm for Cy5‐AFL). The concentrations of inhibitors were: 0.4 m sucrose, 10 µg mL^−1^ chlorpromazine, 80 µm dynasore, 2.5 mm mβ‐cyclodextrin, 0.2 mm genistein, 4 µg mL^−1^ colchicine, 0.5 mm amiloride, and 200 nm wortmannin (Aladdin). Cy5‐AFL or bxyWP‐FAM internalization under uninhibited condition was set as a control (100%).

HCEC were seeded on 4‐chamber glass bottom dishes (Cellvis) at a density of 3 × 10^4^ cells per well and incubated in a humidified incubator at 37 °C with 5% CO_2_ overnight. The Cy5‐AFL/bxyWP‐FAM complexes were pre‐formed by incubating 30 mg mL^−1^ Cy5‐AFL with 0.8 or 3.0 mg mL^−1^ bxyWP‐FAM (containing 0.6 or 2.5 mg mL^−1^ bxyWP). Subsequently, these solutions were diluted to contain 1 µm Cy5‐AFL and incubated with HCEC cells at 37 °C for 2 h before fixation and staining with DAPI. The colocalization was observed using confocal laser scanning microscope (CLSM) (Olympus). The images and M_1_ coefficient were analyzed by ImageJ with Coloc 2 plugin.

### High‐Performance Liquid Chromatography

All the CPPs were quantified by high‐performance liquid chromatography (HPLC) (Agilent) using an ODS column (DiKMA, 250 × 4.6 mm, 5 µm). R_8_ (1.32 mg mL^−1^), PE (2.34 mg mL^−1^), yWP (2.42 mg mL^−1^), xyWP (2.48 mg mL^−1^), and bxyWP (2.54 mg mL^−1^) were incubated with 30 mg mL^−1^ aflibercept. Ultrafiltration was performed to separate the unbound peptides into the lower tube at 14 000 ×g for 10 min. The amount of free peptide was measured by HPLC. The mobile phase was acetonitrile (ACN, Sigma‐Aldrich) containing 0.1% trifluoroacetic acid (TFA, Aladdin): ultrapure water containing 0.1% TFA on a gradient of 5%–65% with a flow rate of 0.7 mL min^−1^ over 30 min. UV absorbance of the effluent was monitored at 280 nm.

To determine the degradation of bxyWP in vitro, 1 mg mL^−1^ bxyWP was incubated with rabbit serum at 37 °C. The incubation was terminated by adding an equal volume of 5% TFA/ACN at 0, 5, 10, 20, and 30 min, 1 and 2 h, respectively. After vortex for 30 s and standing at 4 °C for 5 min, the suspension was centrifuged at 12 000 rpm for 10 min at 4 °C, followed by injection of 25 µL supernatant onto the HPLC system to determine the residual amount of bxyWP. Similarly, 1 mg mL^−1^ bxyWP was incubated with rabbit ocular tissue homogenates at 37 °C and terminated by adding 1.5 times volume of 5% TFA/ACN at 0, 1, 2, 4, 8, 12, and 24 h, respectively. Post‐processing steps were the same as above. The mobile phase was ACN (0.1% TFA): ultrapure water (0.1% TFA) on a gradient of 20%–60% with a flow rate of 0.7 mL min^−1^ over 40 min. UV absorbance of the effluent was monitored at 280 nm.

### Particle Size

Particle sizes of 30 mg mL^−1^ free aflibercept and AFL/bxyWP complexes formed by 30 mg mL^−1^ AFL and 0.6, 1.3, 2.5, 6.4 mg mL^−1^ bxyWP were measured with ZETASIZER Pro (Malvern) by the dynamic light scattering method.

### Structure Modelling and Protein‐Peptide Docking Analysis with Cluspro

The Cluspro 2.0 online server was used to predict the binding site between bxyWP and aflibercept. In the first step, the three‐dimension structure of the bxyWP was modeled using the PEP‐FOLD Peptide Structure Prediction Server (https://mobyle.rpbs.univ‐paris‐diderot.fr/cgi‐bin/portal.py#forms). The three‐dimension structure of aflibercept (Th1133) was obtained from the Therapeutic Protein Database (THPdb, http://crdd.osdd.net/raghava/thpdb/display_thppid_sub.php?details = Th1133). Next, the PDB files for the receptor structure (aflibercept) and the ligand structure (bxyWP) were uploaded onto the Cluspro 2.0 online server (https://cluspro.bu.edu/home.php) to perform automatic docking.^[^ ^64^ ^]^ The most populated clusters of the docking results were then visualized and analyzed using the Chimera software.

### Biolayer Interferometric Assay

Binding kinetics and affinity of protein interaction between aflibercept and bxyWP were studied using the Octet RED96 system (ForteBio). For this measurement, aflibercept at a concentration of 100 µg mL^−1^ was loaded onto Anti‐human IgG Fc Capture (AHC) Biosensors (Sartorius). The association of the analyte, bxyWP, was performed in a three‐fold serial dilution ranging from 0.4 to 33.3 µm in Octet buffer (PBS with 0.02% Tween and 0.1% bovine serum albumin (BSA)). The analyte dissociation was then measured in the Octet buffer. The kinetic cycle, performed at a defined bxyWP concentration, included the following steps: ligand immobilization for 5 min, wash for 5 min, association for 10 min, and dissociation for 10 min. Curve fitting and calculation of association rate constants (k_on_), dissociation rate constants (k_off_), and dissociation constants (K_d_) were performed using Octet data analysis software 10.0. The interaction of aflibercept‐loaded biosensor with no analyte during the association phase was used as the reference sensor.

### Microscale Thermophoresis Assay

Aflibercept solution was first diluted to a 10 mg mL^−1^ stock using a 10 mm 2‐(N‐morpholino)ethanesulfonic acid (MES) buffer with a pH of 6.2. Meanwhile, 10 mg bxyWP was dissolved in 0.9% NaCl solution to prepare a stock of 100 mg mL^−1^. Aflibercept was then labelled with an NHS dye using the Monolith NT Protein Labelling Kit RED‐NHS, following the manufacturer's instructions (NanoTemper Technologies). For the MST experimental setup, an MST buffer with a pH of 5.8 was used, containing 10 mm MES, 150 mm NaCl, and 0.1% PEG 8000. A serial dilution of bxyWP, ranging from 1 mm to 0.03 mm, was mixed with 20 nm labeled aflibercept in a volume ratio of 1:1. The samples were loaded into the Monolith NT.115 instrument (NanoTemper Technologies). To ensure consistency, the assay was performed three times at 25 °C, with the excitation and MST power set to 5% and 40%, respectively. The data obtained was analyzed using the MO. Affinity Analysis software, employing the dissociation constant (K_d_) model with a 1.5 s on‐time.

### In Vitro Transport Across RPE Barrier

ARPE‐19 cells were seeded in Transwell insert chambers (Corning 3470) at a density of 2 × 10^5^ cells cm^−2^ to form a monolayer. The cells were cultured in a 37 °C and 5% CO_2_ incubator. The resistivity of the cell layer was measured using Millicell‐ERS (Millipore) every 2 days. When the transepithelial electrical resistance (TEER) value reached 100 Ω cm^2^ and remained stable,^[^ ^65^ ^]^ the donor sides of in vitro RPE barrier were incubated with 1 µm free aflibercept or AFL/bxyWP complex for 4 h. The acceptor sides were sampled with 200 µL every 0.5 h and immediately replenished with 200 µL fresh D‐Hank's balanced salt solution (Meilun Biotechnology). Aflibercept concentrations in acceptor sides were then measured using enzyme‐linked immunosorbent assay (ELISA). Meanwhile, the donor sides of in vitro RPE barrier were incubated with 5 µm free Cy5‐AFL or Cy5‐AFL/bxyWP complex for 6 h. The samples were then fixed with 4% paraformaldehyde (PFA) (Meilun Biotechnology), stained with DAPI (Beyotime), and observed with CLSM.

### Enzyme‐Linked Immunosorbent Assay

The antigen VEGF_165_ (PeproTech) was pre‐coated on an ELISA plate (Corning Costar 9018). After blocking by 2% BSA (Meilun Biotechnology), 100 µL standards and samples were added. Then, the ELISA plate was shaken at 150 rpm for 1 h at room temperature after sealing. Horseradish peroxidase (HRP)‐conjugated F(ab')2‐goat anti‐human IgG H&L cross‐adsorbed secondary antibody (1:1200) (Abcam, ab81202) was added to the test wells and also shaken at 150 rpm for 1 h at room temperature after sealing. The OD values at 450 and 630 nm were measured immediately with Powerwave XS (BIO‐TEK) after adding chromogenic substrate 3, 3′, 5, 5′‐tetramethylbenzidine (TMB) (Beyotime) and terminating with 2 m H_2_SO_4_ (Sinopharm).

### Immunofluorescence Staining of Tight Junction Proteins

ARPE‐19 cells were seeded on 4‐chamber glass bottom dishes (Cellvis) at a density of 1 × 10^5^ cells per well to form a retinal pigment epithelial layer. The cells were then co‐cultured with 1 µm free aflibercept or AFL/bxyWP complex for 2 h in a 37 °C and 5% CO_2_ incubator. After the incubation, the cells were washed with 1× PBS containing 0.02 mg mL^−1^ heparin. They were then fixed with 4% PFA at room temperature for 15 min, permeabilized with 0.1% Triton X‐100 (diluted in 1× PBS) (Sigma‐Aldrich) at room temperature for 10 min, and blocked with 2% BSA (dissolved in 1× PBS) at room temperature for 1 h. Next, the cells were incubated with primary antibody, either anti‐ZO‐1 tight junction protein antibody (1:100, Abcam, ab221547) or anti‐Occludin antibody (1:100, Abcam, ab216327), at 4 °C overnight. After washing with 1× PBS for 3 times, the cells were further incubated with Alexa Fluor 647 labeled goat anti‐rabbit IgG H&L secondary antibody (1:200, Beyotime, A0468) at room temperature for 1 h in the dark. Finally, the cells were rinsed with 1‰ PBST (PBS with 0.1% Tween 20) for 3 times and stained with DAPI. The tight junction proteins were then imaged using CLSM.

### Western Blotting Analysis of Tight Junction Proteins

ARPE‐19 cells were cultured to form a retinal pigment epithelial layer in a 37 °C, 5% CO_2_ incubator, and then incubated with 1 µm free aflibercept or AFL/bxyWP complex for 2 h. Protein extracts were collected using radio immunoprecipitation assay (RIPA) lysis buffer (Beyotime) containing 1× protease inhibitor cocktail (Beyotime). The concentration of protein was measured using a BCA protein assay kit (Beyotime) and samples were diluted to the same concentration with protein loading buffer (Beyotime). Next, 20 µg protein were separated by SDS‐polyacrylamide gel electrophoresis (Meilun Biotechnology) and electrotransferred to a polyvinylidene difluoride (PVDF) membrane (Merck Millipore). The PVDF transfer membranes were blocked with 5% skim milk powder (Beyotime) at room temperature for 1 h, and then incubated with primary antibodies against ZO‐1 (1:1000, Abcam, ab276131), Occludin (1:1000, Abcam, ab216327), and GAPDH (1:1000, ZSGB‐Bio, TA‐08) at 4 °C overnight. After washing with 1× TBST (tris base solution with 1‰ Tween 20), the membranes were incubated with HRP‐conjugated secondary antibodies (1:5000, ZSGB‐Bio, ZB‐2301&ZB‐2305) at room temperature for 1 h. The signals were visualized using an enhanced chemiluminescence (ECL) reagent (Millipore) and imaged using a FluorChem M system (ProteinSimple). Protein amounts were semi‐quantified using ImageJ and normalized to the amounts of GAPDH.

### Animals

BALB/c nude mice (male, 6–8 weeks), SD rats (male, 160–180 g), and New Zealand white rabbits (male, 2–2.5 kg) were obtained from the Experimental Animal Center of Fudan University. These animals were maintained in a temperature‐ and humidity‐controlled room on a 12 h light‐dark cycle, with free access to water and food. Cynomolgus monkeys (male, 4–6 kg) were supplied by Hua Zhen Laboratory Animal Breeding Centre and individually housed in the animal center of Hua Zhen Biosciences, which maintained a constant ambient temperature (21 ± 5 °C) and humidity (55% ± 15%) on a 12 h light‐dark cycle. The monkeys had free access to water, and commercial feeds were served regularly for breakfast and dinner, with apples provided for lunch. All animals were quarantined one week before the experiment and used according to the guidelines established by Animal Care Committee of the government. All animal experiments were conducted with the permission (No. 2022‐03‐YJ‐WG‐13) of the Ethical Committee of Fudan University.

### IVIS Spectrum CT Imaging

Aflibercept was labeled with Cy5‐NHS (Meilun Biotechnology) as previously described. 3D imaging of BALB/c nude mice body structure and fluorescent signal detection (Ex 640 nm/Em 680 nm, exposure time 0.75 s) in the eye were simultaneously carried out using the IVIS Spectrum CT system (PerkinElmer). The imaging was performed at 5, 10, 20, 30, 45, 60, 120, and 360 min after the topical instillation of 3 µL either free Cy5‐AFL or Cy5‐AFL/bxyWP complex (containing 30 mg mL^−1^ aflibercept). Prior to the imaging, the mice were anesthetized by isoflurane (RWD Life Science) to ensure their immobilization during the detection process.

### Intraocular Distribution

Two drops of 5 µL free Cy5‐AFL or Cy5‐AFL/bxyWP complex (containing 30 mg mL^−1^ Cy5‐AFL) were instilled into the conjunctival sac of rats at a 3 min interval. The eye drops were administered 3 times a day at a 4 h interval. The rats were sacrificed 1 h after the last administration, and the eyes were collected to prepare DAPI‐stained frozen sections. These sections were then observed under CLSM. The fluorescence intensity of Cy5‐AFL from corneal epithelium to endothelium and from conjunctiva to inner retina was plotted using ImageJ software.

Two drops of 50 µL free aflibercept or AFL/bxyWP complexes were instilled into the conjunctival sac of rabbits at a 3 min interval. The AFL/bxyWP complexes were pre‐formed by incubating 30 mg mL^−1^ aflibercept with 1.3 or 2.5 mg mL^−1^ bxyWP, or 60 mg mL^−1^ aflibercept with 2.5 or 5.1 mg mL^−1^ bxyWP. Additionally, two drops of 30 µL AFL/bxyWP (1.3 mg mL^−1^) complex or two drops of 50 µL free aflibercept and AFL/bxyWP (2.5 mg mL^−1^) complex were instilled into the conjunctival sac of rabbits at a 3 min interval, administered 3 times a day at a 4 h interval. An hour after the last administration, the rabbits were anesthetized and euthanatized, and blood was immediately collected from the heart to obtain serum. The eyes were enucleated, rinsed, and dissected into cornea, conjunctiva, sclera, aqueous humor, vitreous humor, and retina‐choroid. Each tissue was weighed and lysed, and the concentrations of aflibercept were determined using ELISA assay.

### In Vivo Treatment of Laser‐Induced CNV

CNV lesions were bilaterally induced in male cynomolgus monkeys using a 532 nm laser system (Vitra laser photocoagulator, Quantel Medical). Six lesions were placed in an orderly manner around the macula of both eyes. The specific laser parameters were as follows: laser power of 800 mW, duration of 0.1 s, and spot size of 50 µm.

Seven days after laser injury, CNV lesions were evaluated by fundus fluorescein angiography (FFA), and 13 monkeys were randomly divided into three groups. From day 8, monkeys in the negative control (NS) group received topical administration of normal saline, while monkeys in the eye drops (Eye drops) group received AFL/bxyWP complex (containing 30 mg mL^−1^ aflibercept) three times a day (50 µL per drop, two drops at a 3 min interval) for 2 weeks. The positive control (IVT) group received a single intravitreal injection of 40 mg mL^−1^ aflibercept (50 µL per eye) in both eyes on day 8, administrated by a specialist after anesthesia and sanitizing with povidone iodine. Ofloxacin eye ointment (Santen) was applied twice a day for 3 days.

FFA was performed on day 7, 15, and 22 after the CNV modelling using a Heidelberg Multi‐color OCT. After anesthetizing the monkeys and dilating their pupils, 10% fluorescein sodium solution (10 mg kg^−1^, Alcon) was administered via rapid intravenous injection, and the angiograms were obtained within 10 min. The lesion grades were defined as follows: grade 1, no hyperfluorescence, grade 2, hyperfluorescence without leakage, grade 3, early hyperfluorescence and late mild leakage within the border of the burn area, grade 4, early hyperfluorescence and late severe dye leakage beyond the border of the burn area.^[^ ^66^ ^]^

Optical coherence tomography (OCT) was performed using the Heidelberg SPECTRALIS‐plus system to evaluate the thickness of the retina and the presence of subretinal hyperreflective material (SHRM) on day 7, 15, and 22 after the CNV modelling. The scanning area covered all the laser coagulation spots and the anterior corneal tissues.

### Safety Profiles

On day 0, 7, 15, and 22, ophthalmic examinations were conducted using a slit lamp. Additionally, OCT (Heidelberg) and rebound tonometer (iCare) were used to monitor the changes in corneal thickness and intraocular pressure, respectively. Before modeling and after the treatment, all test animals were fasted overnight, and their blood samples were collected in the following morning before breakfast for hematology and biochemical examinations.

### Statistical Analysis

Statistical analyses were performed using GraphPad Prism 8 software and IBM SPSS 25 software. In experiments comparing a single experimental group to a single control group, statistical comparisons were made by two‐tailed unpaired *t*‐test. In experiments comparing multiple experimental groups to a control group, multiple comparisons were made by one‐way ANOVA corrected by Dunnett's test, while in experiments comparing each experimental group with every other experimental group, one‐way ANOVA with multiple comparisons corrected by Tukey's test was used to determine the statistical significance. For comparisons of experiments with two variables, two‐way ANOVA with multiple comparisons corrected by Sidak's test was employed. Regression analysis was performed to test the relationship between the cellular uptake of aflibercept and the binding affinity between aflibercept and CPPs. The threshold for statistical significance was set at *P *< 0.05.

## Conflict of Interest

Gang Wei, the corresponding author of this manuscript, has a part‐time position as a consultant at Alephoson Biopharmaceuticals Limited. This outside activity may be considered a potential conflict of interest in relation to the work reported in this paper. The authors declare that they have no other competing interests.

## Author Contributions

G.W., W.L., B.L., X.F., K.J., and Y.Z. contributed to the study conception and experimental design. X.F. performed most experiments, collected and interpreted data. Y.Z. assisted in performing structure modelling, protein‐peptide docking analysis, biolayer interferometric and microscale thermophoresis experiments. F.G. assisted in performing experiments with mice and rabbits and drew the schematic diagrams. G.W., X.F., and K.J. contributed to preparing the figures. X.F., M.E.B., and Y.Z. cowrote the original manuscript. G.W., X.F., K.J., Y.Z., and M.E.B. revised the manuscript. G.W., W.L., and B.L. contributed to project administration. G.W. supervised the project and acquired funding. All authors contributed to critical review of the manuscript.

## Supporting information



Supporting Information

Supplemental Video 1

Supplemental Video 2

Supplemental Video 3

## Data Availability

The data that support the findings of this study are available from the corresponding author upon reasonable request.
