# Correction to “Peptide‐Bound Aflibercept Eye Drops for Treatment of Neovascular Age‐Related Macular Degeneration in Nonhuman Primates”

**DOI:** 10.1002/advs.202519902

**Published:** 2025-11-14

Fan X, Jiang K, Zhao Y, Lee BT, Geng F, Brelen ME, Lu W, Wei G. Peptide‐Bound Aflibercept Eye Drops for Treatment of Neovascular Age‐Related Macular Degeneration in Nonhuman Primates.

*Adv Sci* (Weinh). 2025 Mar;12(11):e2410744.

https://doi.org/10.1002/advs.202410744

We have identified an unintentional error in Figure 6C of our published article. Specifically, the labels for b2 and b3 were inadvertently miswritten. The corrected image is presented below.


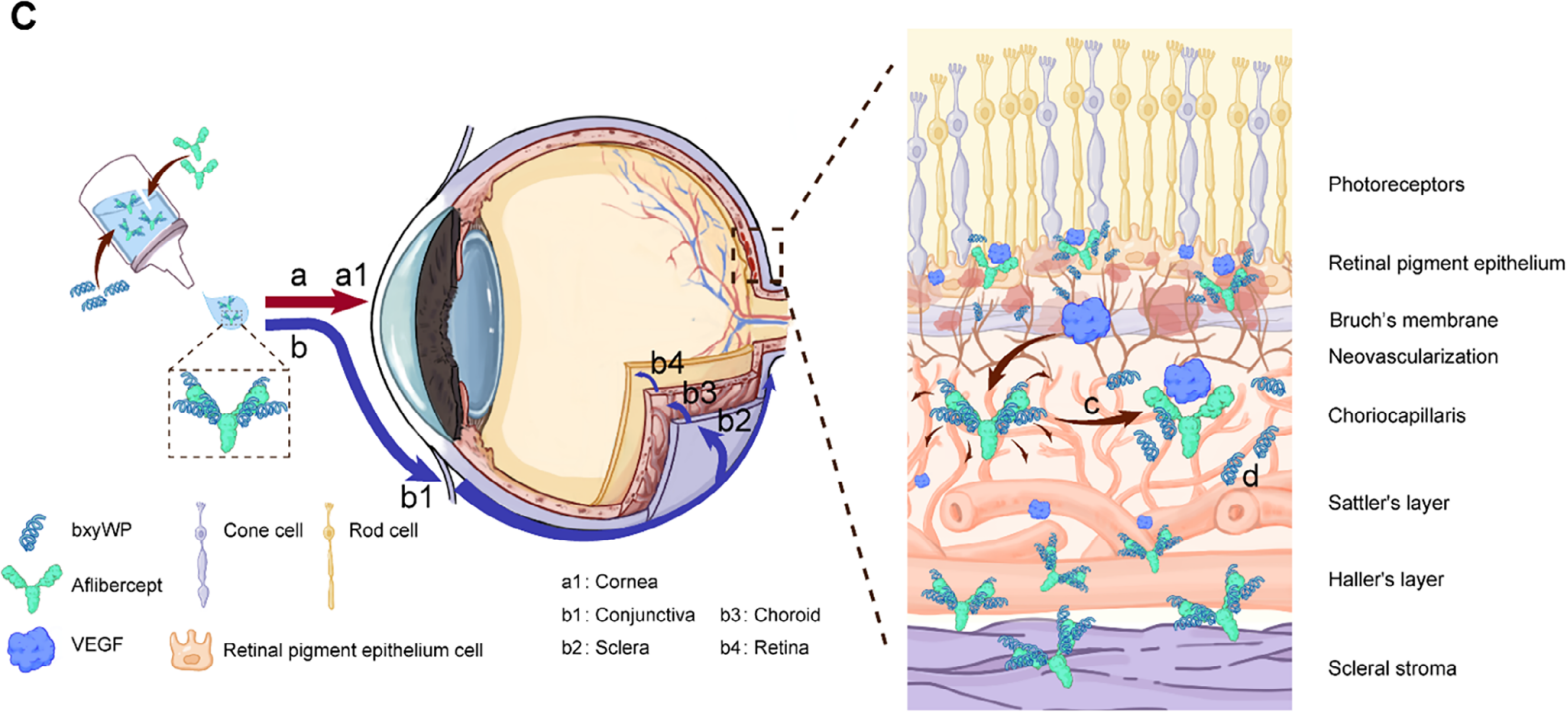


**Figure 6**(C). Schematic diagram of the ocular absorption pathway of the AFL/bxyWP complex: a) corneal pathway; b) conjunctival‐scleral pathway; c) binding of aflibercept to VEGF; d) released bxyWP would be degraded in serum or ocular tissues.

We declare that this correction does not affect the results, interpretation, or conclusions of this study.

We apologize for this error.

