# Supplementary material for: Peptide‐Bound Aflibercept Eye Drops for Treatment of Neovascular Age‐Related Macular Degeneration in Nonhuman Primates
Source: Adv Sci (Weinh). 2025 Jan 30;12(11):2410744. doi: 10.1002/advs.202410744 (PMC11923875; doi:10.1002/advs.202410744)
Supplement: Supplementary file 1 — Supporting Information [file ADVS-12-2410744-s004.docx]

Supporting Information

**Peptide-bound Aflibercept Eye Drops for Treatment of Neovascular Age-related Macular Degeneration in Nonhuman Primates**

*Xingyan Fan^1^, Kuan Jiang^2^, Yongqian Zhao^3^, Benjamin TK Lee^3^, Feiyang Geng^1^,* *Marten E Brelen^4^, Weiyue Lu^1, 5^, Gang Wei^1, 5, 6^**

^1^Department of Pharmaceutics, School of Pharmacy, Fudan University & Key Laboratory of Smart Drug Delivery (Fudan University), Ministry of Education, Shanghai, 201203, China

^2^Eye Institute and Department of Ophthalmology, Eye and ENT Hospital, Fudan University, Shanghai, 200031, China

^3^Alephoson Biopharmaceuticals Limited, Hong Kong SAR, 999077, China

^4^Department of Ophthalmology and Visual Sciences, The Chinese University of Hong Kong, Hong Kong SAR, 999077, China

^5^Quzhou Fudan Institute, Quzhou, 324003, China

^6^Shanghai Engineering Research Center of ImmunoTherapeutics, Shanghai, 201203, China

*Correspondence to [weigang@shmu.edu.cn](mailto:weigang@shmu.edu.cn)

**Table S1. The sequences and miLogP of R_8_ and penetratin derivatives**

| Peptide | Sequence | miLogP ^a^ |
| --- | --- | --- |
| R_8_ | RRRRRRRR | -6.41 |
| Penetratin | RQIKIWFQNRRMKWKK | -6.01 |
| bWP | R**W**IKIWFQNRRMKWKK | -5.57 |
| xWP | RQIKIWF**W**NRRMKWKK | -5.57 |
| yWP | RQIKIWFQ**W**RRMKWKK | -5.75 |
| byWP | R**W**IKIWF**W**NRRMKWKK | -4.76 |
| byWP | R**W**IKIWFQ**W**RRMKWKK | -5.13 |
| xyWP | RQIKIWF**WW**RRMKWKK | -5.13 |
| bxyWP | R**W**IKIWF**WW**RRMKWKK | -3.40 |

^a^ Molecular hydrophobicity, calculated by Molinspiration (http://www.molinspiration.com/cgi/properties)

**Table S2. Particle size and polydispersity index (PDI) of 30 mg/mL AFL and AFL/bxyWP complexes incubated at 4 °C for 24 h.**

| AFL (mg/mL) | bxyWP (mg/mL) | Particle size (d. nm) | PDI |
| --- | --- | --- | --- |
| 30 | 0 | 13.85 ± 0.08 | 0.110 ± 0.013 |
| 30 | 0.6 | 15.73 ± 0.10 | 0.117 ± 0.007 |
| 30 | 1.3 | 18.16 ± 0.20 | 0.156 ± 0.009 |
| 30 | 2.5 | 22.63 ± 0.41 | 0.156 ± 0.010 |
| 30 | 6.4 | 34.95 ± 0.66 | 0.250 ± 0.015 |

**
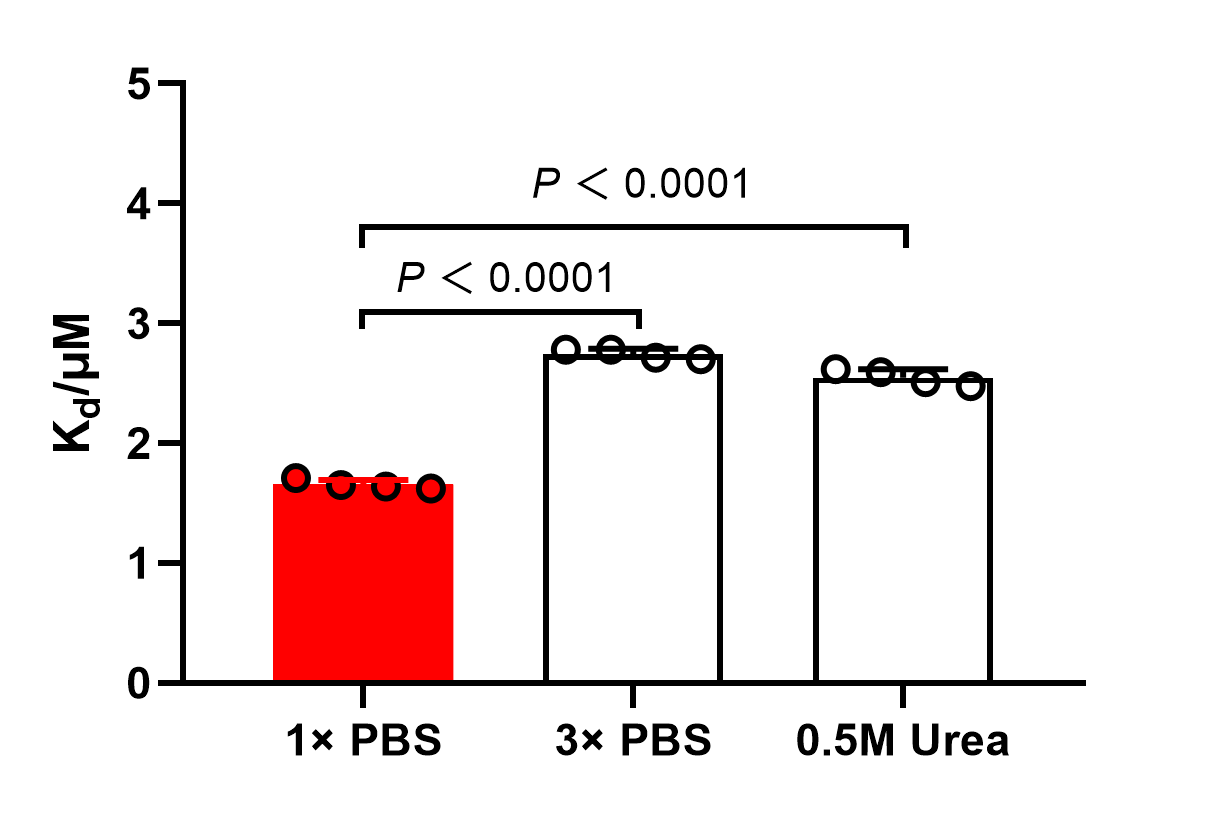
**

**Figure S1. Determination of K_d_ for AFL/bxyWP complex to understand the type of non-covalent interactions.** Equilibrium fluorescence quenching assay was performed at 25 ℃ in 3× PBS, or in 1× PBS with the addition of 0.5 M urea (n = 4). Data are presented as means ± SD. Statistical analysis was performed using one-way ANOVA with multiple comparisons corrected by Dunnett’s test.

**
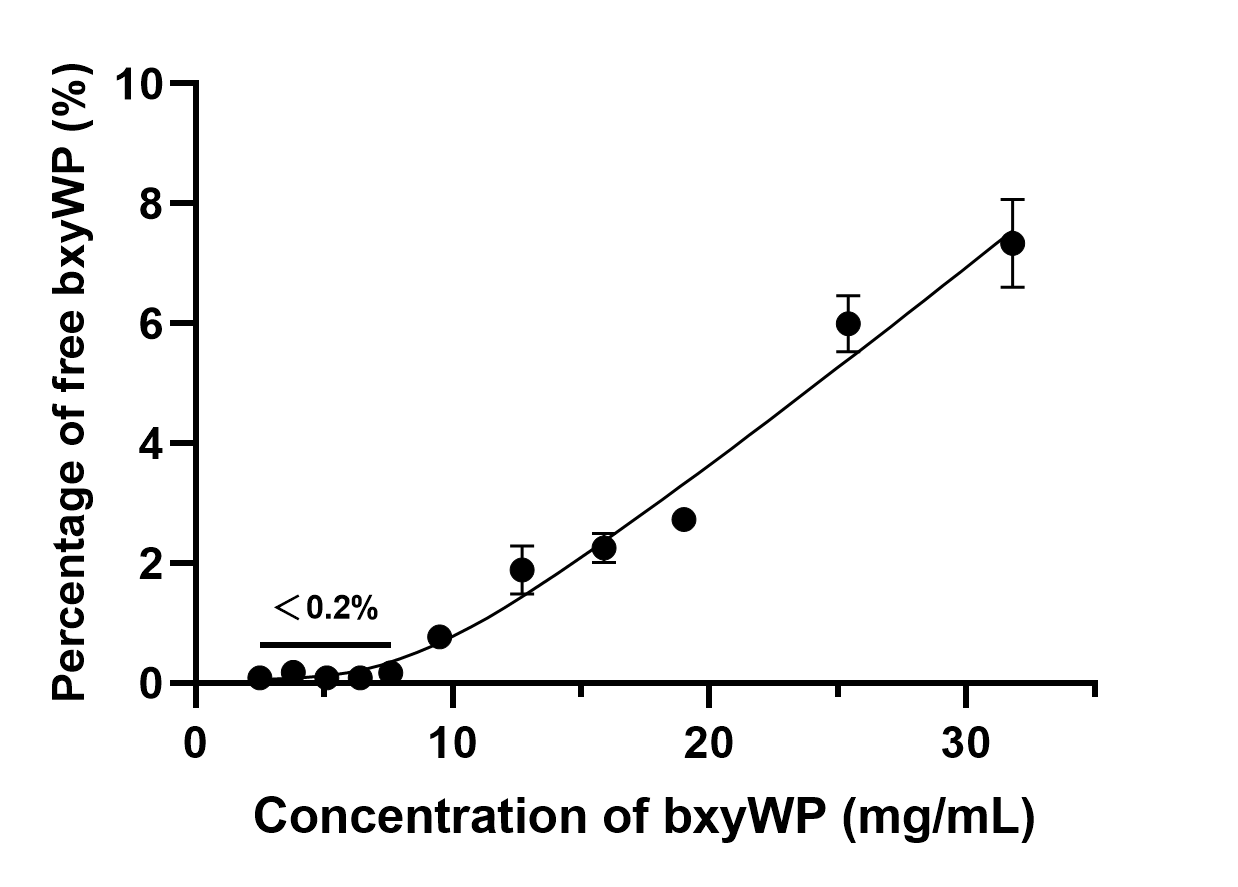
**

**Figure S2. Determination of the amount of free bxyWP in various AFL/bxyWP complexes using ultrafiltration.** The AFL/bxyWP complexes were pre-formed by incubating 30 mg/mL aflibercept with 2.5, 3.8, 5.1, 6.4, 7.6, 9.5, 12.7, 15.9, 19.0, 25.4, and 31.8 mg/mL bxyWP (n = 3). Data are presented as means ± SD.


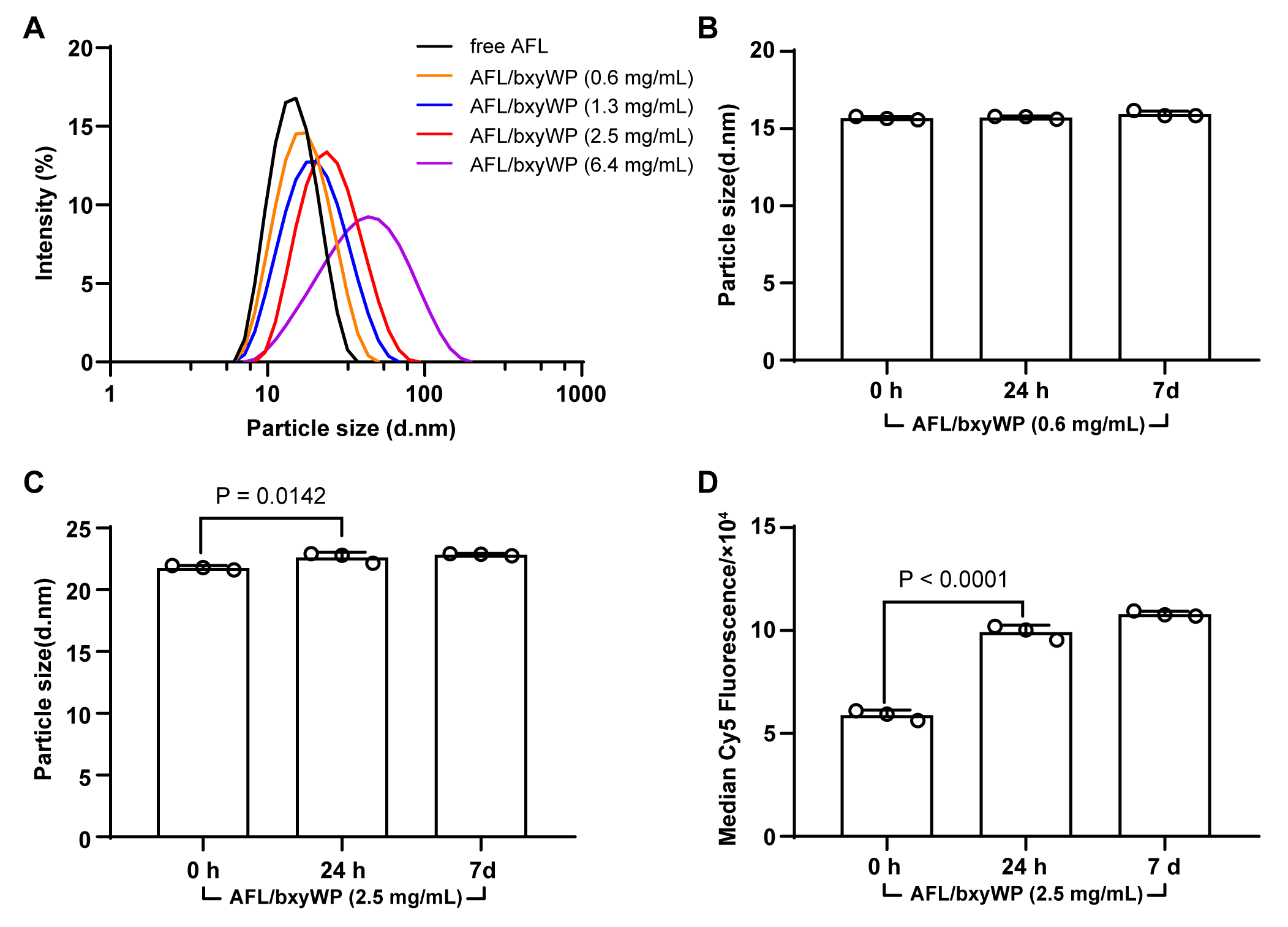


**Figure S3. Particle size and cellular uptake of various AFL/bxyWP complexes. (A)** Size distribution of free aflibercept and AFL/bxyWP complexes formed by 30 mg/mL AFL and 0.6, 1.3, 2.5, 6.4 mg/mL bxyWP. **(B**, **C)** Particle size of AFL/bxyWP (0.6 mg/mL) and AFL/bxyWP (2.5 mg/mL) complexes, respectively incubated at 4°C for 0 hour, 24 hours and 7 days (n = 3). **(D)** Flow cytometry analysis of the median fluorescence intensity in ARPE-19 cells treated with AFL/bxyWP (2.5 mg/mL) complex after mixing for different time points (n = 3). Aflibercept (30 mg/mL) and bxyWP (2.5 mg/mL) were mixed and incubated at 4°C for 0 hour, 24 hours and 7 days. These solutions were then diluted to contain 1 μM Cy5-labelled aflibercept and incubated with ARPE-19 cells for 2 hours at 37 °C before detection by the flow cytometry (Ex 633 nm/Em 660 nm). Data are presented as means ± SD. Statistical analysis was performed using one-way ANOVA with multiple comparisons corrected by Dunnett’s test.


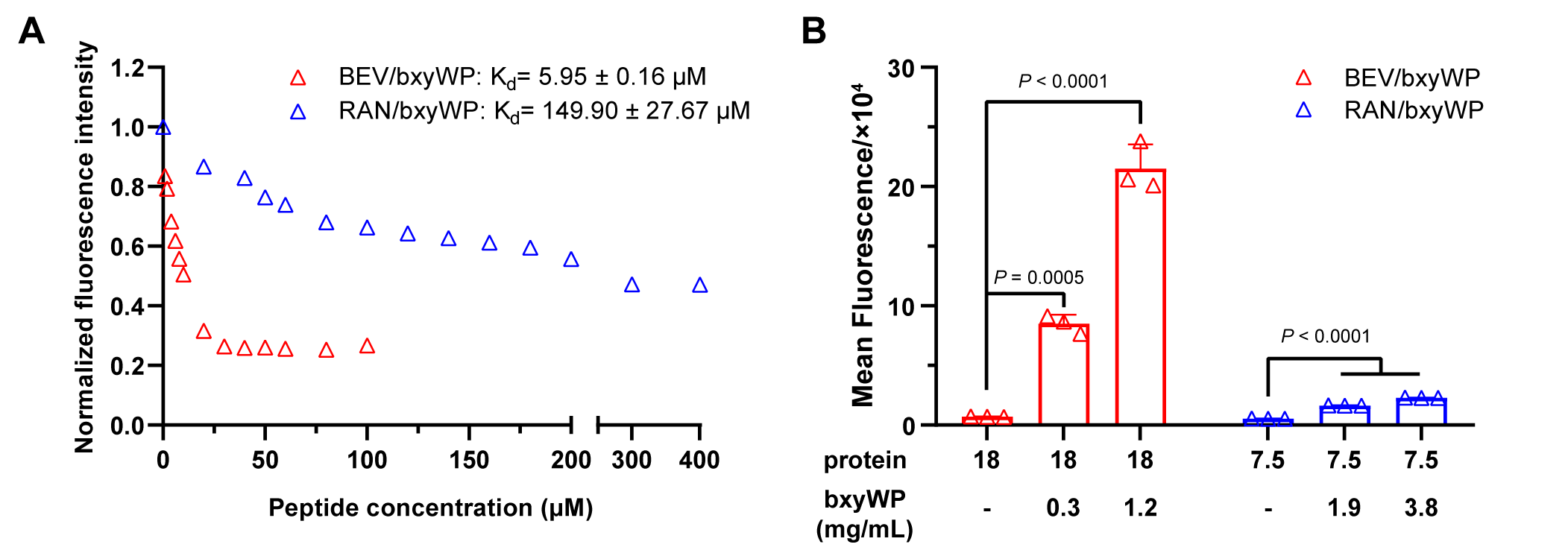


**Figure S4. The interactions between different anti-VEGF proteins and bxyWP.** (**A**) Binding curves of bxyWP with bevacizumab (BEV) and ranibizumab (RAN). The K_d_ for BEV/bxyWP and RAN/bxyWP complexes were determined using equilibrium fluorescence quenching assay at 25 ℃ in 1× PBS (n = 4). (**B**) Flow cytometry analysis of the mean fluorescence intensity of BEV/bxyWP and RAN/bxyWP complexes in ARPE-19 cells. The BEV/bxyWP and RAN/bxyWP complexes (each containing 1 μM BEV or RAN) were incubated with the cells for 2 hours at 37 °C. Data are presented as means ± SD. Statistical analysis was performed using one-way ANOVA with multiple comparisons corrected by Dunnett’s test (n = 3).


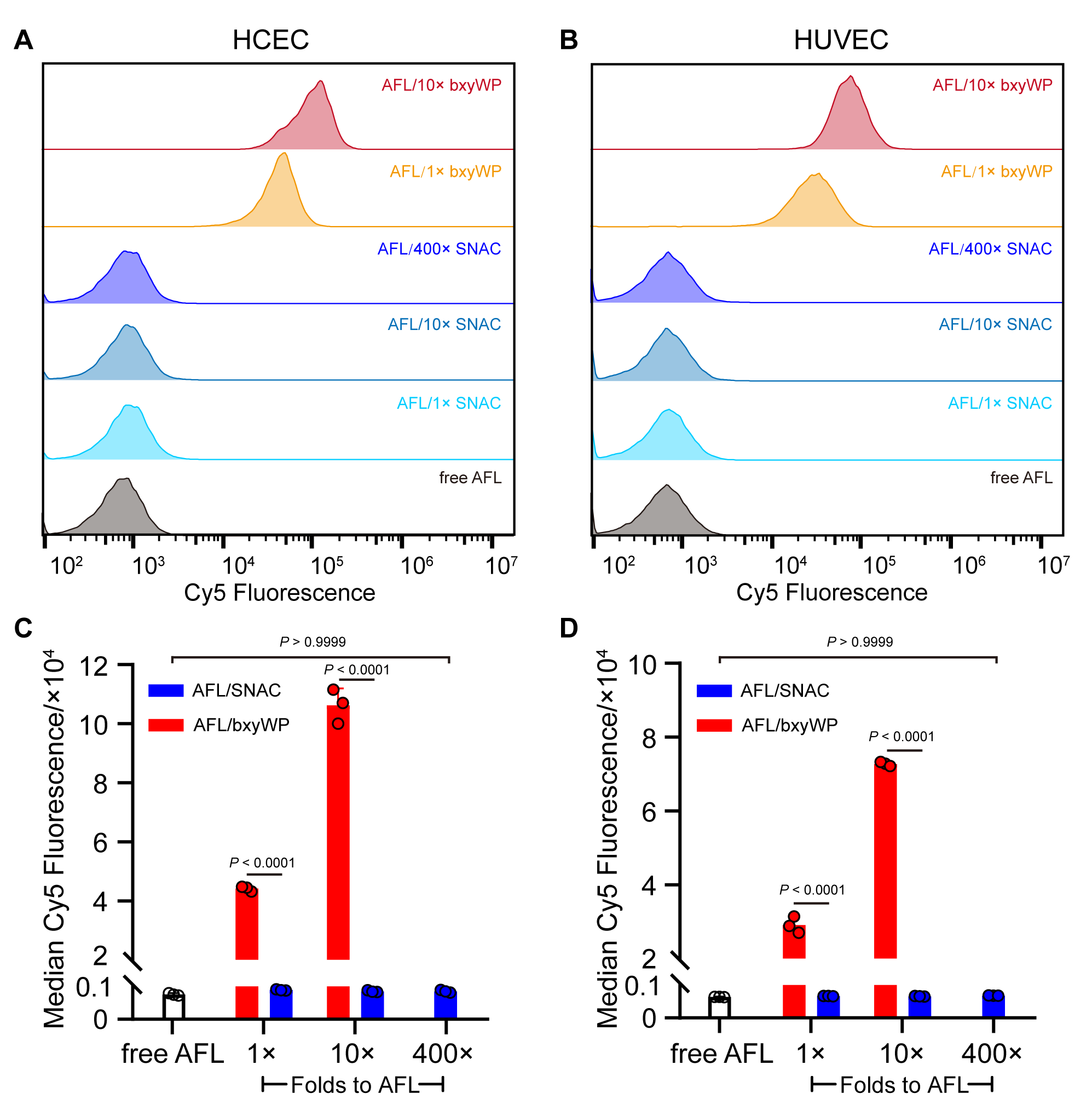


**Figure S5. Comparison of the ability of SNAC and bxyWP to promote the cellular uptake of aflibercept in HCEC and HUVEC cells.** The cells were incubated with Cy5-labelled free aflibercept and various complexes for 2 hours at 37 °C (final concentration of aflibercept=1 μM). Data are presented as means ± SD. Statistical analysis was performed using one-way ANOVA with multiple comparisons corrected by Sidak’s test (n = 3).


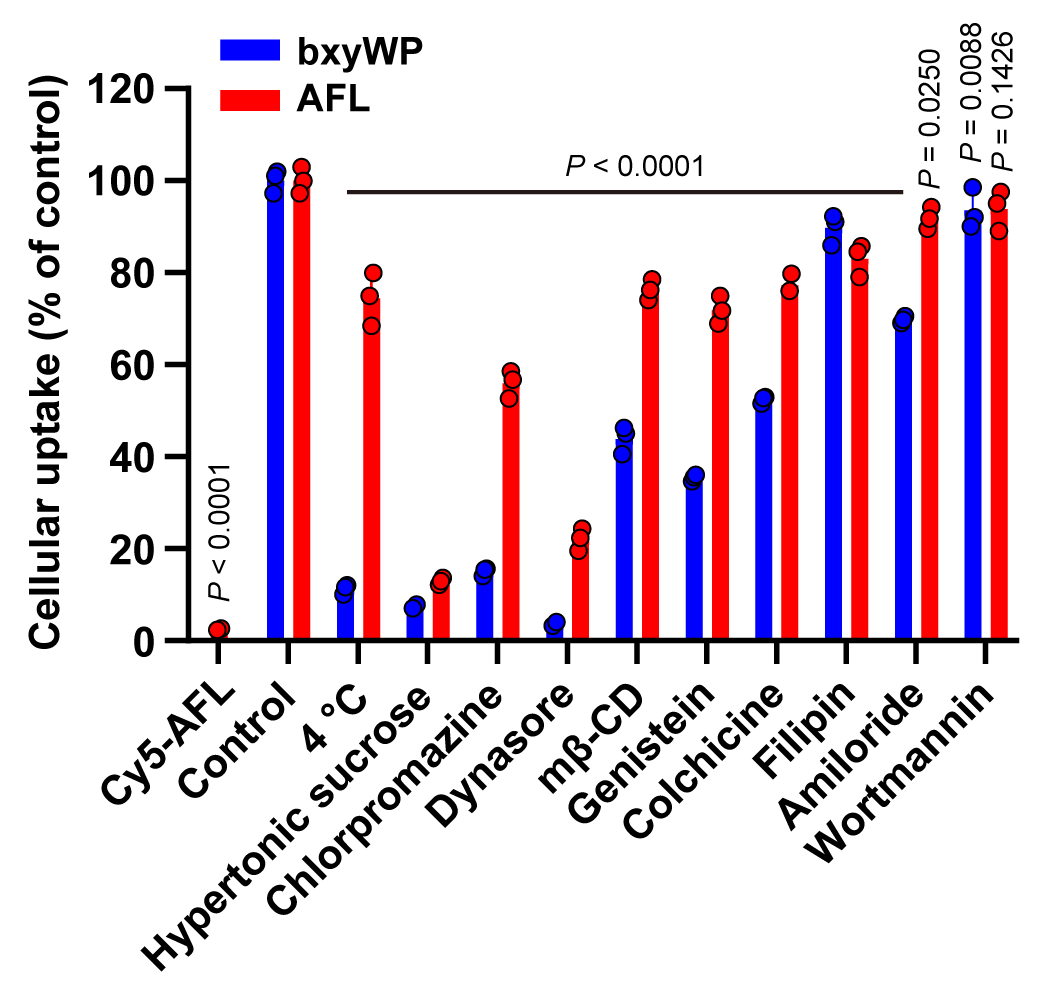


**Figure S6. Effect of temperature and endocytosis inhibitors on the cellular uptake of AFL/bxyWP complex in ARPE-19 cells.** The complexes were pre-formed by incubating 30 mg/mL Cy5-labelled aflibercept and 7.6 mg/mL FAM-labelled bxyWP, and then were diluted to contain 1 μM aflibercept. The complexes were incubated with ARPE-19 cells for 2 hours at 37 °C before detection of the cells by the flow cytometry (Ex 633 nm/Em 660 nm). The internalization of FAM-labelled bxyWP or Cy5-labelled aflibercept under uninhibited condition was set as the control (100%). Data are presented as means ± SD. Statistical analysis was performed using one-way ANOVA with multiple comparisons corrected by Dunnett’s test compared with the control group (n = 3).


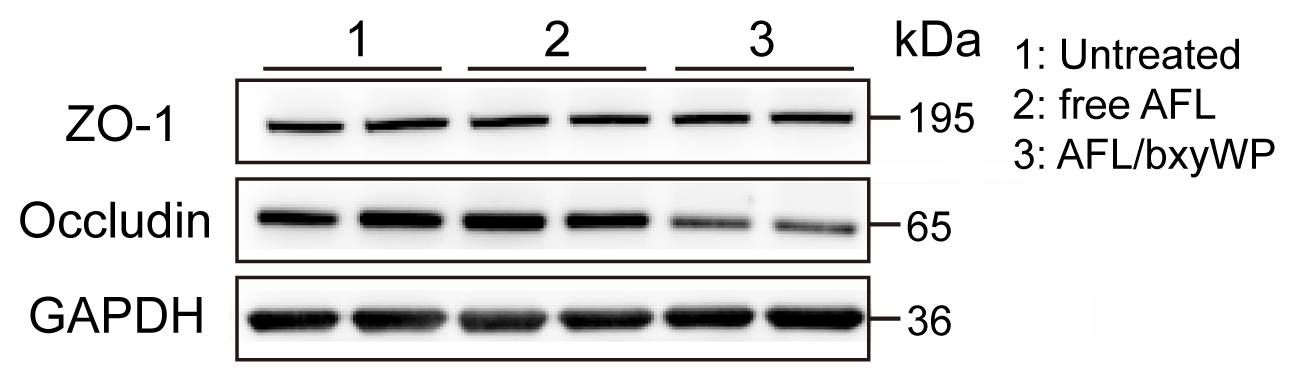


**Figure S7. Western blotting images showing the expression of ZO-1 and occludin in ARPE-19 monolayer.** The cells were incubated with either free aflibercept or AFL/bxyWP complex for 2 hours before lysis and protein extraction.


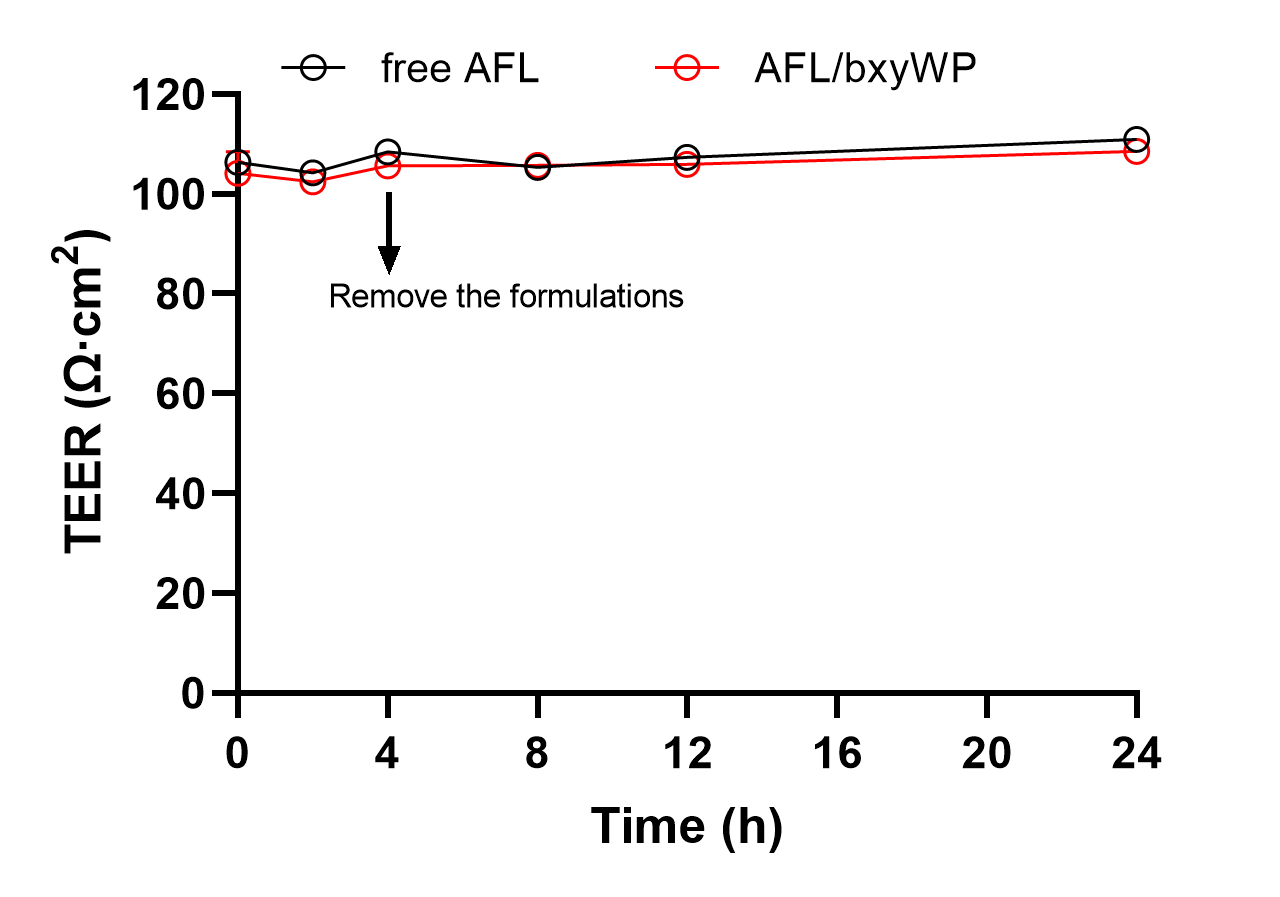


**Figure S8. Measurement of transepithelial electrical resistance (TEER) in 24 hours.** Data are presented as means ± SD (n = 3).


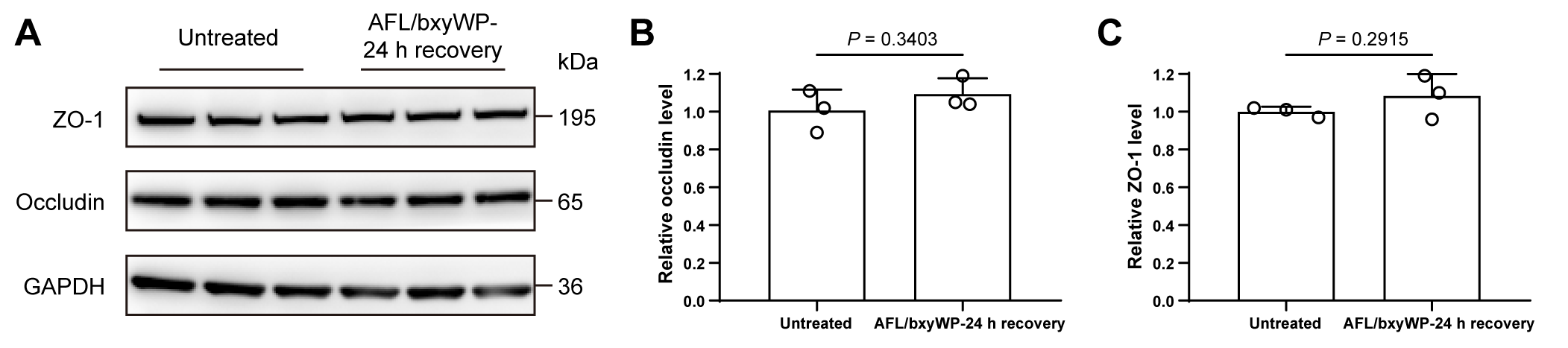


**Figure S9. Western blotting images (A) and statistical analysis of occludin (B) and ZO-1 (C) expression levels in ARPE-19 monolayer after incubation with AFL/bxyWP complex for 2 hours and then recovery for 24 hours.** Data are presented as means ± SD. Statistical analysis was performed using two-tailed unpaired *t*-test (n = 3).


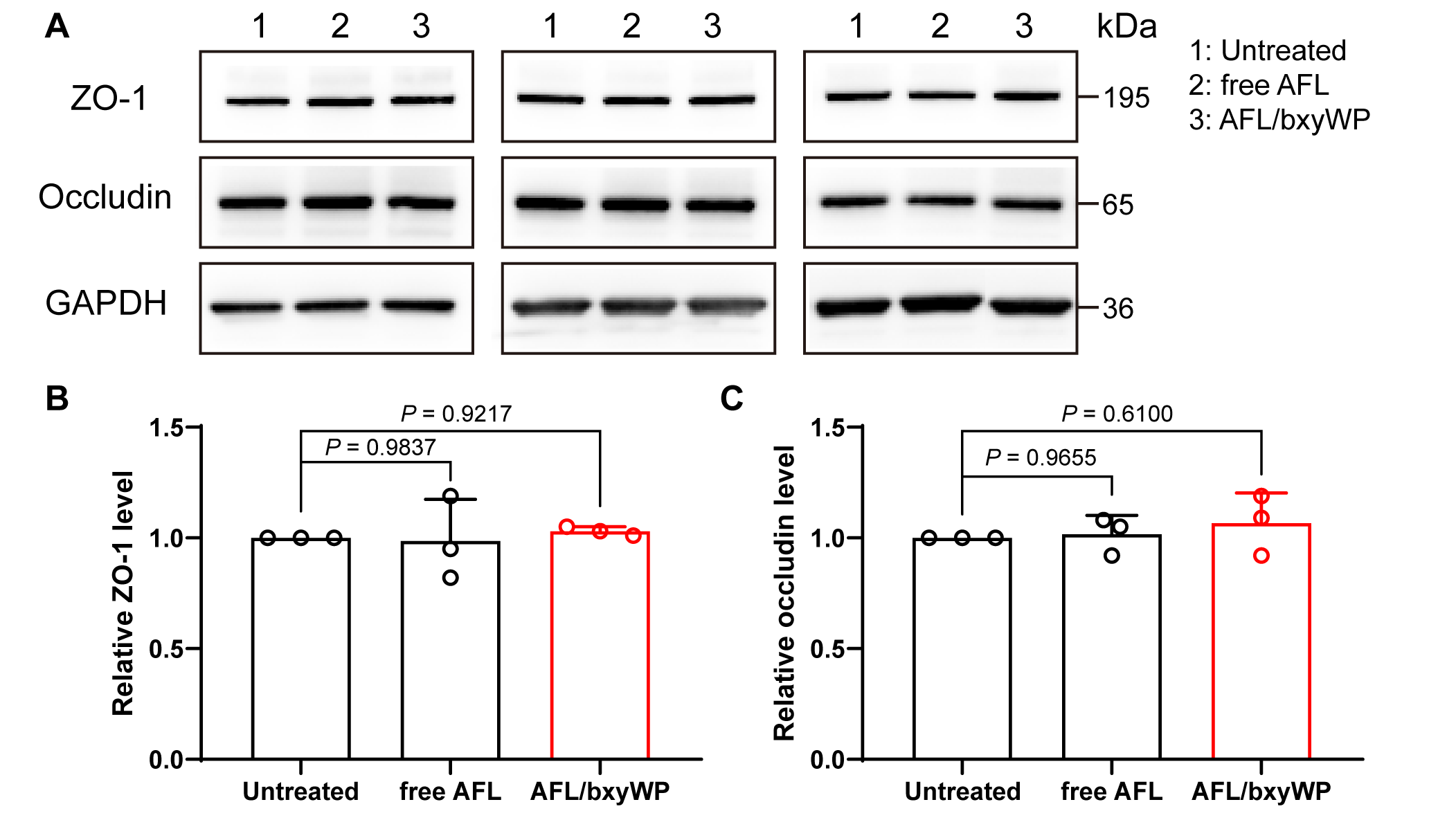


**Figure S10. Western blotting images and statistical analysis of occludin and ZO-1 expression level in** **HCEC monolayer.** The cells were incubated with either free aflibercept or AFL/bxyWP complex for 2 hours before lysis and protein extraction. Data are presented as means ± SD. Statistical analysis was performed using one-way ANOVA with multiple comparisons corrected by Dunnett’s test (n = 3).


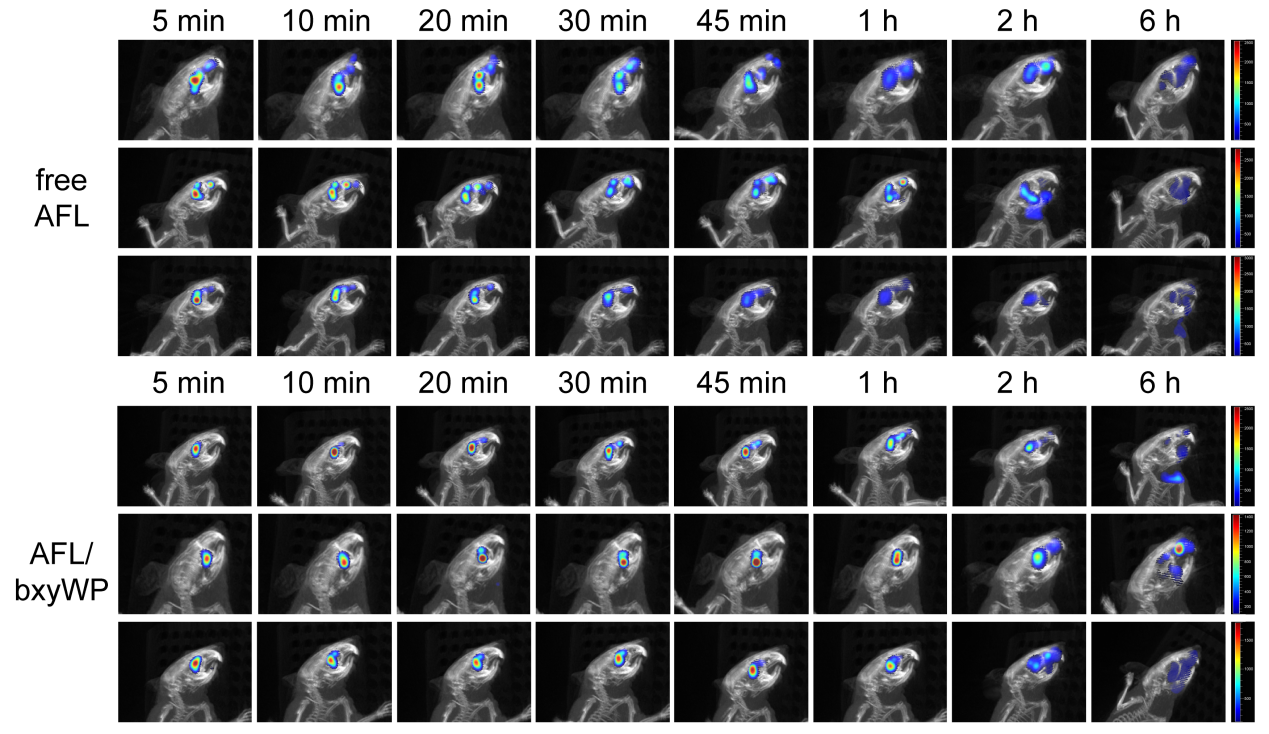


**Figure S11. Retention of AFL/bxyWP complex in mouse eyes.** IVIS Spectrum CT images of Cy5-labelled aflibercept in the eyes after topical instillation of either free aflibercept or AFL/bxyWP complex (30 mg/mL aflibercept, 3 μL/eye) at different time points (n = 3).


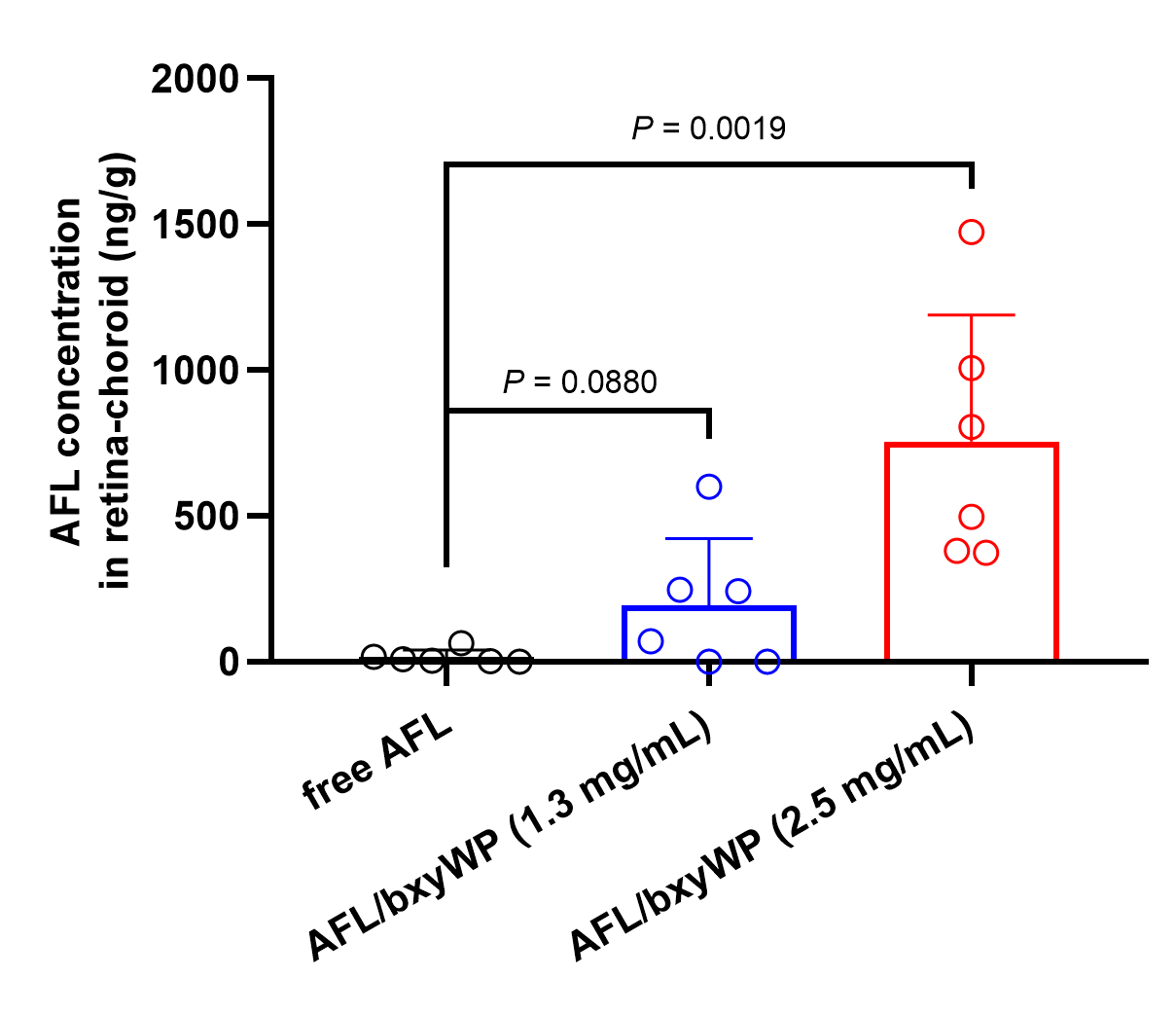


**Figure S12.** **Concentration of aflibercept in retina-choroid at 1 hour after the last dose of topical instillation of free aflibercept and AFL/bxyWP** **complexes.** All the eye drops contained 30 mg/mL aflibercept and were administered 3 times a day at a 4-hour interval via topical instillation. The volume of each drop was 30 μL for AFL/bxyWP (1.3 mg/mL) complex and 50 μL for free aflibercept and AFL/bxyWP (2.5 mg/mL) complex, and two drops were administered each time (n = 6). Data are presented as means ± SD. Statistical analysis was performed using two-tailed unpaired *t*-test.


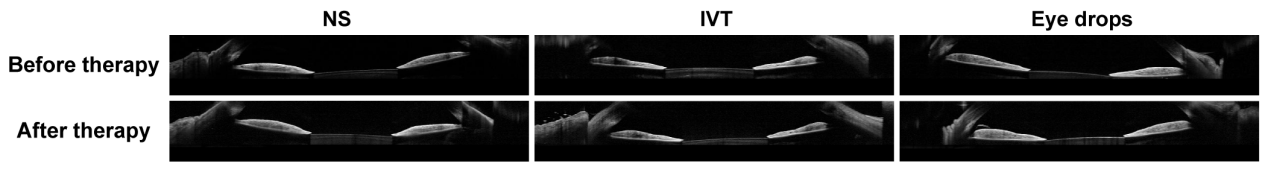


**Figure S13. Representative OCT images of anterior chamber angle in the different treatment groups.** NS, normal saline; IVT, intravitreal injection.


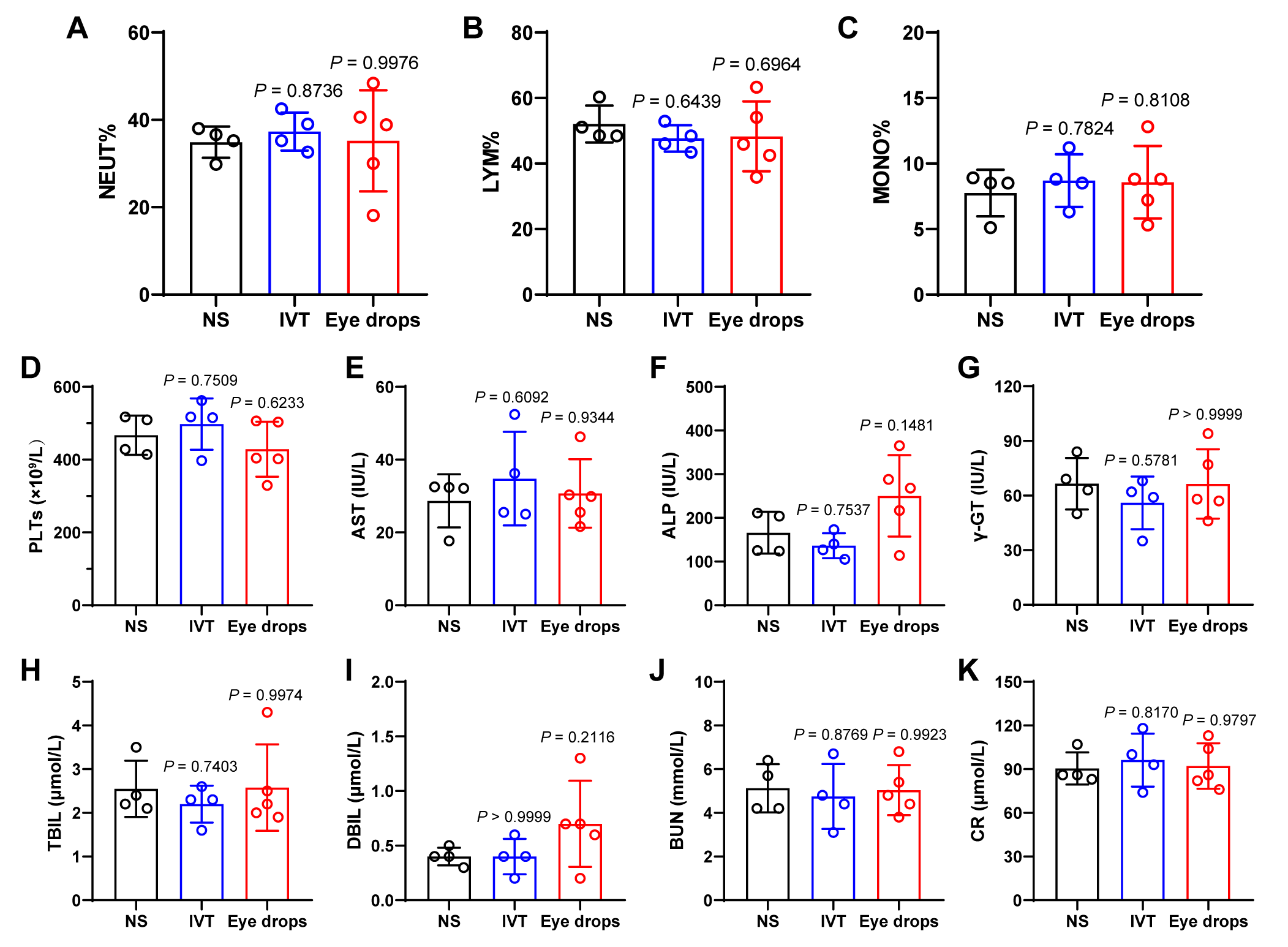


**Figure S14. Blood routine and blood biochemistry examinations of monkeys after therapy.** n = 4 for NS and IVT groups, n = 5 for Eye drops group. Data are presented as means ± SD. Statistical analysis was performed using one-way ANOVA with multiple comparisons corrected by Dunnett’s test compared with the NS group. Notes: NEUT, neutrophil; LYM, lymphocyte; MONO, monocyte; PLT, platelet; AST, aspartate aminotransferase; ALP, alkaline phosphatase; γ-GT, γ-glutamyl transferase; TBIL, total bilirubin; DBIL, direct bilirubin; BUN, blood urea nitrogen; CR, creatinine; NS, normal saline; IVT, intravitreal injection.


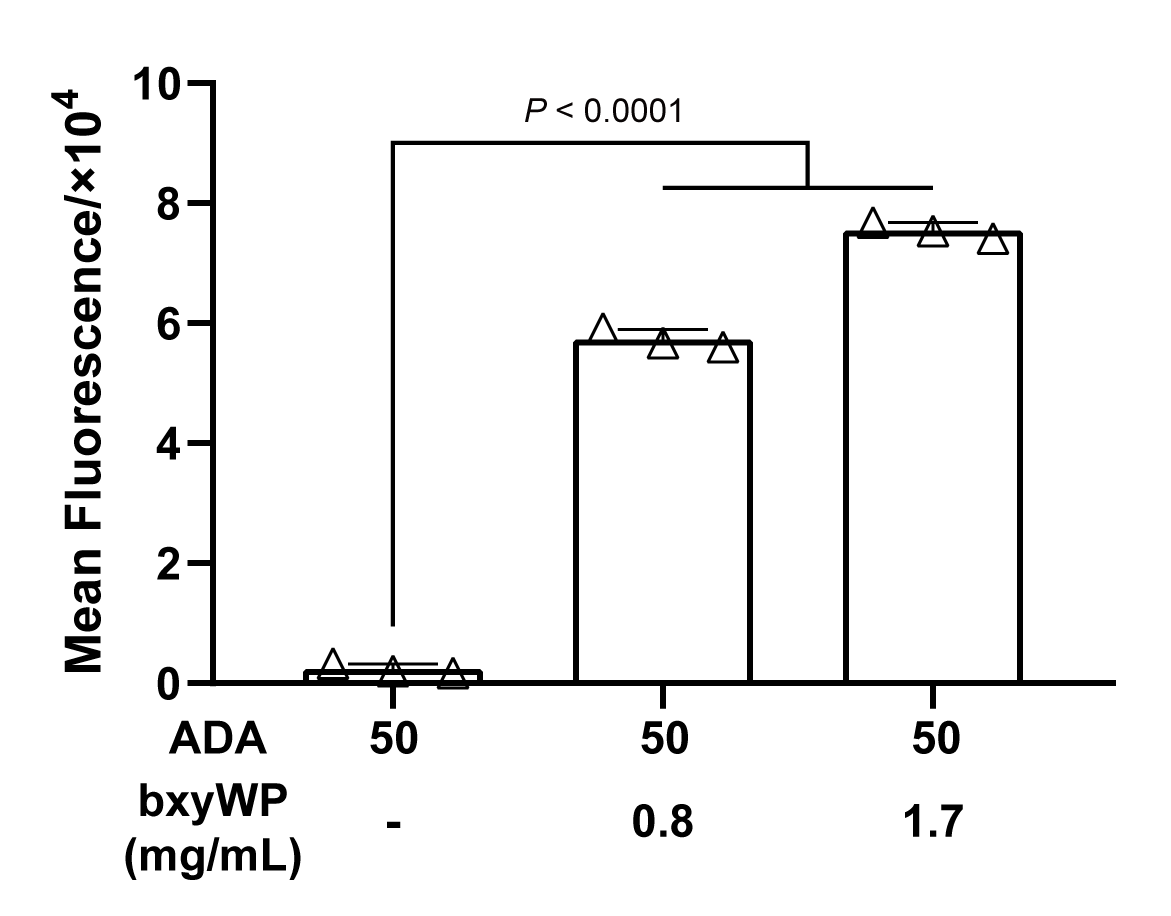


**Figure S15. Flow cytometry analysis of the mean fluorescence intensity of Adalimumab/bxyWP (ADA/bxyWP) complex in ARPE-19 cells.** The ADA/bxyWP complex (each containing 1 μM Cy5-labelled ADA) was incubated with the cells for 2 hours at 37 °C. Data are presented as means ± SD. Statistical analysis was performed using one-way ANOVA with multiple comparisons corrected by Dunnett’s test (n = 3).

**Supplementary video 1. Binding sites of bxyWP on aflibercept.** The rotating video, obtained using the Cluspro 2.0 server demonstrates the potential binding sites of the bxyWP peptide on the aflibercept protein. Aflibercept is displayed in electrostatic surface potentials, colored red (-) and blue (+), while bxyWP is shown as a cartoon in various colors.

**Supplementary video 2. Three-dimensional demonstration of the retention of free aflibercept in mouse eyes.** IVIS Spectrum CT imaging video depicts the retention of Cy5-labelled aflibercept in the eye after topical instillation of free aflibercept (30 mg/mL aflibercept, 3 μL/eye) at different time points (n = 3).

**Supplementary video 3. Three-dimensional demonstration of the retention of AFL/bxyWP complex in mouse eyes.** IVIS Spectrum CT imaging video depicts the retention of Cy5-labelled aflibercept in the eye after topical instillation of AFL/bxyWP (2.5 mg/mL) complex (30 mg/mL aflibercept, 3 μL/eye) at different time points (n = 3).
